# T2 mapping in myocardial disease: a comprehensive review

**DOI:** 10.1186/s12968-022-00866-0

**Published:** 2022-06-06

**Authors:** Aaron T. O’Brien, Katarzyna E. Gil, Juliet Varghese, Orlando P. Simonetti, Karolina M. Zareba

**Affiliations:** 1 Ohio University Heritage College of Osteopathic Medicine, Athens, Ohio USA; 2 Department of Internal Medicine, Division of Cardiovascular Medicine, The Ohio State University Wexner Medical Center, Columbus, Ohio USA; 3 Dorothy M. Davis Heart and Lung Research Institute, The Ohio State University, Columbus, Ohio USA; 4 Department of Radiology, The Ohio State University, Columbus, Ohio USA

**Keywords:** T2 Mapping, Myocardial edema, Myocardial inflammation, Parametric mapping, Cardiomyopathy

## Abstract

Cardiovascular magnetic resonance (CMR) is considered the gold standard imaging modality for myocardial tissue characterization. Elevated transverse relaxation time (T2) is specific for increased myocardial water content, increased free water, and is used as an index of myocardial edema. The strengths of quantitative T2 mapping lie in the accurate characterization of myocardial edema, and the early detection of reversible myocardial disease without the use of contrast agents or ionizing radiation. Quantitative T2 mapping overcomes the limitations of T2-weighted imaging for reliable assessment of diffuse myocardial edema and can be used to diagnose, stage, and monitor myocardial injury. Strong evidence supports the clinical use of T2 mapping in acute myocardial infarction, myocarditis, heart transplant rejection, and dilated cardiomyopathy. Accumulating data support the utility of T2 mapping for the assessment of other cardiomyopathies, rheumatologic conditions with cardiac involvement, and monitoring for cancer therapy-related cardiac injury. Importantly, elevated T2 relaxation time may be the first sign of myocardial injury in many diseases and oftentimes precedes symptoms, changes in ejection fraction, and irreversible myocardial remodeling. This comprehensive review discusses the technical considerations and clinical roles of myocardial T2 mapping with an emphasis on expanding the impact of this unique, noninvasive tissue parameter.

## Background

Decay of transverse magnetization, marked by the relaxation time constant T2, has long been known as an index of myocardial edema. Cardiovascular magnetic resonance (CMR) studies from the early 1980s in canines demonstrated a positive linear relationship between myocardial water content and T2 relaxation time in myocardial ischemia [1, 2]. These ex vivo investigations using spin echo pulse sequences provided an early framework for the use of T2 relaxation time in the detection of myocardial edema. Subsequent in vivo canine studies used electrocardiogram (ECG) gated spin echo pulse sequences to assess elevated myocardial T2 times after coronary artery occlusion and reperfusion [3, 4]. The first CMR study establishing feasibility, safety, and utility of the technique for human patients with acute myocardial infarction (AMI) took place in 1989 [5]. These early in vivo studies were limited by imaging artifacts caused by blood flow and breathing motion. These limitations were overcome by a pulse sequence that combined turbo spin echo with short-inversion-time inversion-recovery (STIR), and blood signal nulling [6] that enabled T2-weighted imaging of the heart within a breath-hold. This form of T2-weighted imaging (T2w-STIR) remains one of the predominant CMR modes for assessment of myocardial edema.

**Fig. 1:**
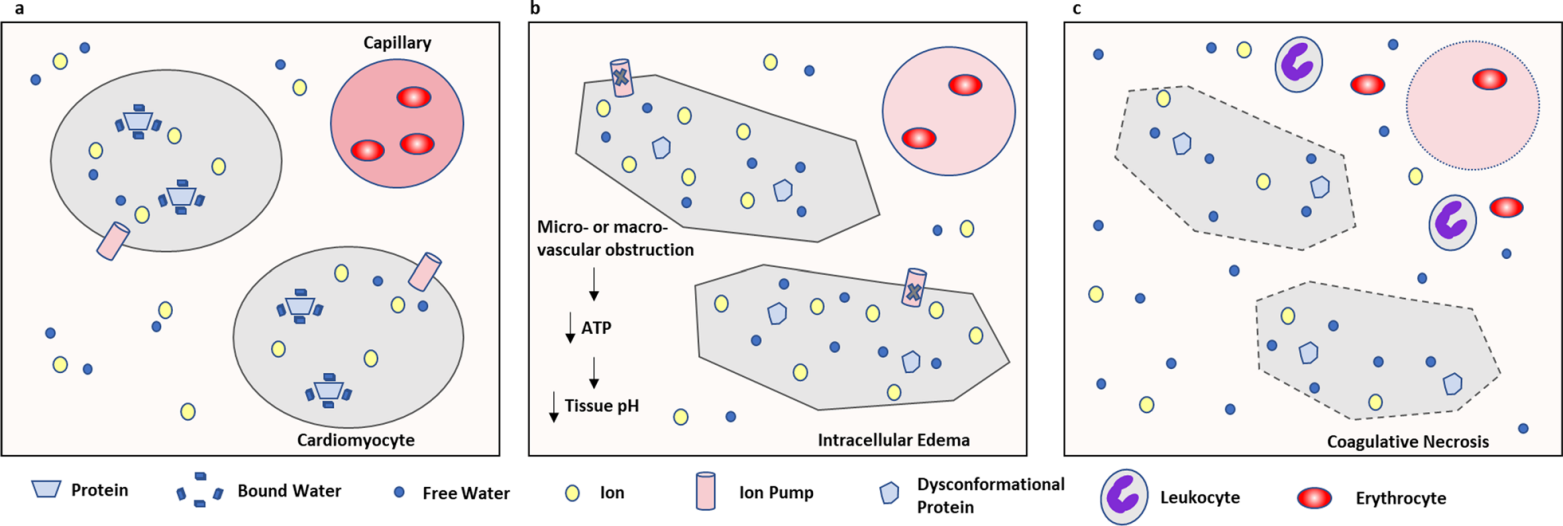
Pathophysiology of Changes in Free Water and Water Content During Ischemic Myocardial Injury. A simplified graphical representation of (**a)** equilibrium of free water and bound water during steady physiologic state. During ischemia due to micro- or macro-vascular obstruction, (**b)** adenosine triphosphate (ATP)-dependent ion pumps are disrupted resulting in fluid shifting intracellularly and an increase in free water relative to bound water without change in net water content. Decreased intracellular pH changes protein conformation—favoring release of bound water. Finally, reperfusion of necrotic myocardium through damaged microvasculature (**c**) results in leakage of intravascular fluid and cells into the interstitial space with gain of free water in this compartment and net water increase. Adapted with permission from Springer Nature: Nature Reviews Cardiology. Friedrich MG. Myocardial edema–a new clinical entity? Nat Rev Cardiol. 2010;7(5):292–296. https://doi.org/10.1038/nrcardio.2010.28

Quantitative T2 mapping overcomes several limitations of T2-weighted (T2w) imaging and results in more accurate evaluation of myocardial edema [7]. T2 mapping has been validated in an animal study by showing strong correlation with actual myocardial water content [8]. It is important to note that myocardial T2 time may be elevated by changes in myocardial water state, and not only net water content (Fig. 1). This explains why T2 relaxation time may be elevated in myocardial diseases that do not produce changes in gross water content. Examples of conditions that may elevate T2 times without changing net water content include changes in the fraction of free water vs. water bound to macromolecules, fluid shifts between extracellular and intracellular compartments, intracellular vacuolization, changes in collagen content, and orientation of collagen fibrils [9–15]. Thus, T2 relaxation time is a versatile index for a range of myocardial pathologies.

**Fig. 2:**
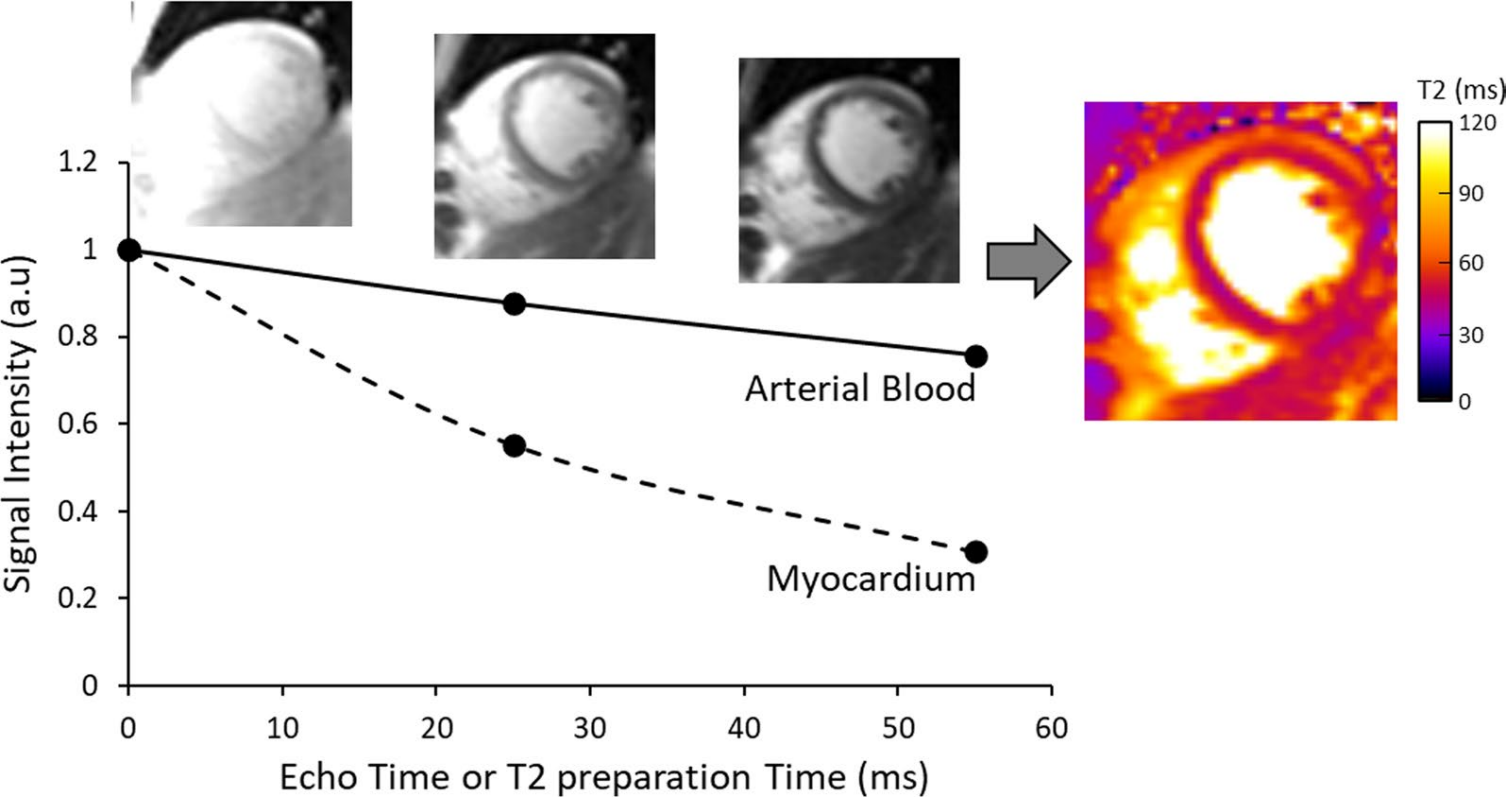
Quantitative Determination of Myocardial T2 Relaxation Time. In order to generate quantitative maps of myocardial T2 relaxation time, several images with varying T2 sensitivity are acquired at the same cardiac phase over multiple cardiac cycles. For each pixel location, the signal intensities across the T2 weighted (T2w) images are then fit to a mono-exponential decay equation to estimate the T2 relaxation time and generate a T2 map. These maps are often displayed using a color scale for easier visualization. The graph demonstrates the transverse signal decay of myocardium and arterial blood, as measured from the three single-shot T2 prepared balanced steady state free precession (bSSFP) images acquired at T2 preparation times of 0, 25 and 55 ms. Since myocardial T2 relaxation time is approximately four times shorter than arterial blood, this difference is observed in the faster signal decay in the myocardium across the T2w images and in the color map. Placing a region of interest in the map provides the user with the corresponding T2 relaxation time

T2 mapping differs from T2w imaging in that it renders pixel-wise T2 times for myocardium on a continuous millisecond (ms) scale. The basic requirements for any conventional T2 mapping method are: (a) acquisition of a series of T2w images at varying T2 sensitivity; (b) co-registration of the multiple source images; (c) estimation of T2 relaxation time by performing a pixel-wise fit of the T2w signal to a mono-exponential decay equation (Eq. 1.), where $S\left(x,y\right)$ is the acquired signal intensity for the pixel location $\left(x,y\right)$, ${M}_{0}(x,y)$ is the steady-state signal, ${TE}_{T2p}$ is the T2 preparation time, $T2$ is the T2 relaxation time, and $C$ is a constant offset that may or may not be used based on the fitting model; and (d) reconstruction of a quantitative parametric map, usually color coded for easier visualization (Fig. 2).

1 $$S(x,y)= {M}_{0}(x,y){e}^{\frac{-{TE}_{T2p}}{T2(x,y)}}+C$$

T2 mapping provides reliable quantitative information about focal, regional, patchy, or diffuse myocardial disease, whereas T2w imaging relies on semi-quantitative comparison of signal intensities between normal and diseased myocardium, or between myocardium and skeletal muscle. Depending on the pulse sequence, advantages of T2 mapping over T2w imaging include reduced sensitivity to motion, reduced signal intensity variability, improved detection of endocardial borders, and increased objectivity [7].

This comprehensive review discusses T2 mapping from two perspectives. The technical considerations of T2 mapping are discussed first, followed by established and emerging clinical roles as well as future directions for this CMR imaging modality.

## T2 map acquisition

**Fig. 3:**
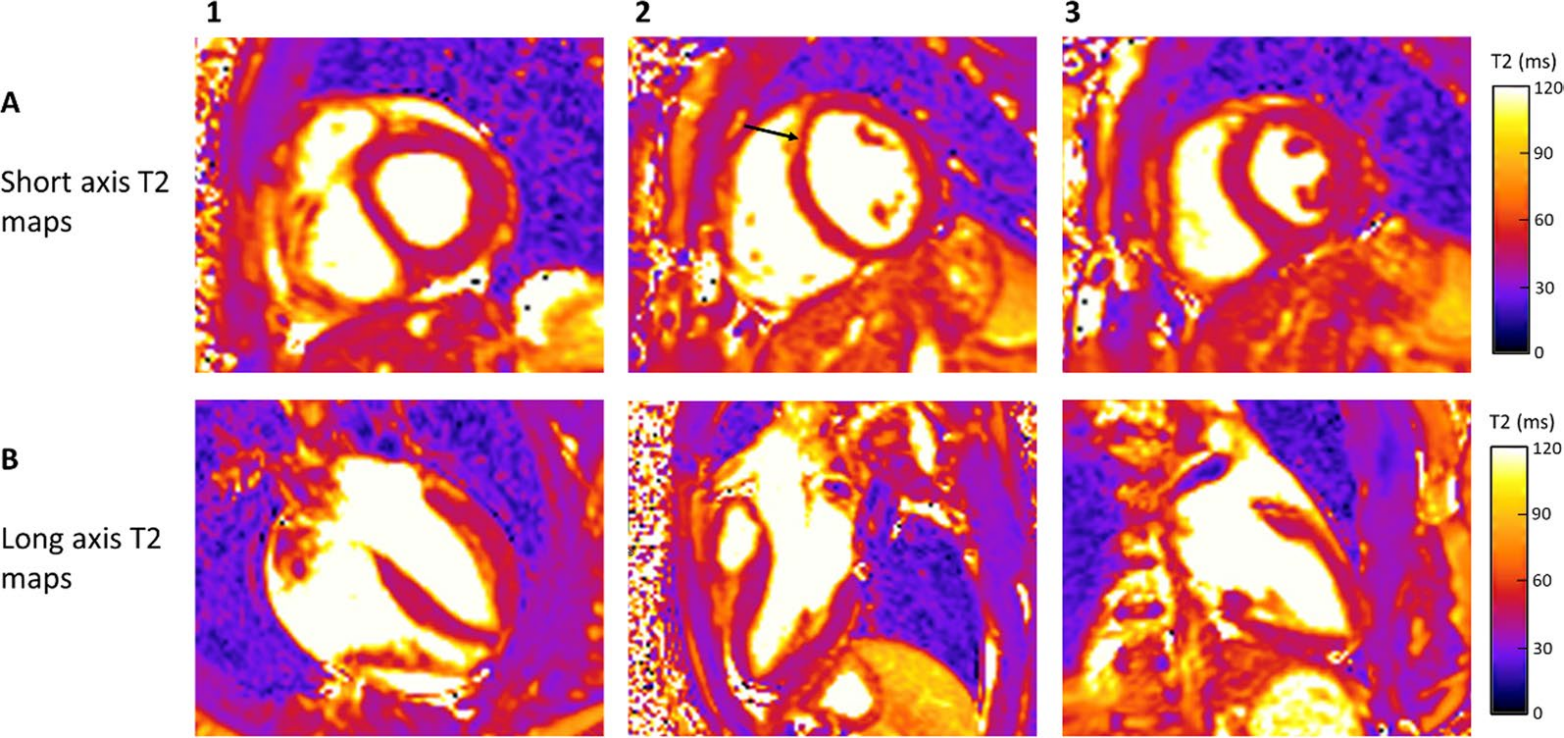
T2 Mapping in a Patient with no Evidence of Myocardial Edema/Inflammation. Short axis (Panel **A1**–**A3**) and long axis T2 maps (Panel **B1**–**B3**). Focal T2 time prolongation in the mid anteroseptal wall on the map acquired during diastole (Panel **A2**; black arrow), most likely caused by partial volume artifact, is no longer present on the map acquired during systole (Panel **A3**). **A1**—basal short axis view; **A2**—midmyocardial short axis view acquired during diastole; **A3**—midmyocardial short axis view acquired during systole; **B1**—horizontal long axis view; **B2**—three-chamber view; **B3**—vertical long axis view. T2-prepared bSSFP pulse sequence with the use of rectangular T2 preparation pulse utilized to acquire T2 short axis (SAx) map during diastole (Panel **A2**) and with adiabatic T2 preparation pulse to acquire long axis and SAx T2 maps during systole (Panel **A1**, **A3**, **B1–B3**)(1.5 T MAGNETOM Sola, Siemens Healthineers)

T2 maps may be acquired in any imaging plane, typically in the vertical long axis (VLA or two-chamber), horizontal long axis (HLA or four-chamber), three-chamber, and short axis (SAx) planes (Fig. 3). Mapping can be performed at field strengths (B0) of 1.5 T, or 3 T. Source images may be acquired with or without magnetization preparation, in a single-shot or segmented fashion, ECG-triggered with breath-holding, or as navigator-gated free breathing scans, in 2D or 3D, with Cartesian or radial readouts, and combined with non-rigid motion correction as needed. These acquisition methods are briefly summarized below.

### B0 field strength

T2 relaxation times decrease with increasing field strength. Myocardial T2 maps are commonly acquired at 1.5 T and 3 T [7, 16–20] with feasibility demonstrated at lower field strengths [21, 22] as well. Imaging at higher field strength provides a signal-to-noise ratio (SNR) gain that may afford higher spatial and temporal resolution. On the other hand, increased B0 and B1 inhomogeneities experienced at higher field strengths can create non-uniform T2 preparation, resulting in artifacts that confound image interpretation. Use of B1-insensitive adiabatic T2 preparation pulses, application of B0 and B1 shimming, and use of gradient recalled echo (GRE) instead of balanced steady state free precession (bSSFP) acquisition modules can mitigate these artifacts to some extent and ensure successful imaging at 3 T [23, 24].

### T2 preparation

Magnetization preparation based T2 mapping utilizes a T2 preparation module to create T2 contrast, followed by a readout or data acquisition module using a fast sequence such as bSSFP [25] or GRE. This allows the T2w magnetization to be sampled before bSSFP steady-state is reached and creates image contrast that is primarily due to the T2 relaxation time. The T2 preparation module duration, or T2 preparation time (T2prep), is adjusted to acquire images at varying echo times. The T2 preparation module consists of a 90° excitation or tip-down pulse to tip the magnetization into the transverse plane, a train of equally spaced 180° pulses to refocus the transverse magnetization, followed by a 90° tip-up pulse to restore the T2w magnetization to the longitudinal axis. This T2 preparation scheme was first used to achieve muscle and venous suppression for improved visualization of coronary arteries [26]. Combinations of single or composite rectangular hard pulses ordered with Malcolm-Levitt phase cycling [27] and adiabatic pulses have been implemented in the T2 preparation module to achieve motion and flow insensitivity, and B0 and B1 homogeneity. Adiabatic pulse designs such as B1-insensitive refocusing [23], modified B1-insensitive rotation [28] and Silver-Hoult [29], provide even greater B0 and B1 homogeneity than rectangular pulses.

The T2 preparation module can be non-selective [26] for single-slice imaging or slice-selective for multi-slice imaging [30]. Mapping techniques that do not utilize magnetization preparation are usually multi-echo spin echo (MESE) based methods where multiple T2-weighted images with different echo times are acquired to generate a series of images with varying T2 sensitivity.

### Single shot or segmented acquisition

T2 maps can be generated from a series of single-shot T2w source images [7, 25]. These source images can also be acquired in a segmented fashion to improve temporal resolution, or for 3D volumetric imaging, at the expense of longer acquisition times [31–34]. The benefits of single-shot 2D imaging are that the k-space lines for a single T2 prepared source image are acquired in a single heartbeat, and all source images are acquired within a single breath-hold. Single-shot techniques may however suffer from both fast and variable heart rates. Unless adjusted, the wait time between T2 prepared images is shortened with higher heart rates; this can introduce T1 weighting (T1w) into the signal as a result of the incomplete magnetization recovery, leading to underestimated T2 relaxation times. Variable heart rates could create variable T1 influence between source images, leading to altered signal behavior and errors in the estimated T2. Non-selective 90° saturation pre-pulses have been used prior to the acquisition module to reset the magnetization and avoid this heart rate dependency [35, 36].

### Motion compensation

Single-shot mapping methods employ ECG-triggering and breath-holds to compensate for cardiac and respiratory motion. However, poor breath-hold compliance in patients can result in mis-registration of the source images, causing mapping errors and the need for repeated acquisitions. The use of non-rigid motion correction algorithms can avoid spatial mis-registration of the source images, thereby improving the accuracy of the pixel-wise T2 fit and the subsequent quality of the maps; it cannot however account for through-plane motion arising from different breath-hold positions. Navigator-gating has been utilized in both 2D and 3D T2 mapping to provide respiratory motion compensation that allows images to be acquired free-breathing, and to remove constraints on achievable spatial and temporal resolution [16, 37, 38]. However, the reduced acquisition efficiency can prolong scan times. Scanning efficiency has been improved with image-based navigator respiratory motion correction methods [39, 40].

### 2D or 3D

Several 2D single-slice T2 maps at different imaging planes can be easily included in the routine CMR workflow as each map can be acquired in a short breath-hold lasting 7–10 s. However, repetitive breath-holds and the cumulative scan time become limiting factors when extensive myocardial tissue characterization or whole heart coverage is desired. In addition, the thicker 2D maps (usually 8–10 mm) provide non-isotropic resolution, which may introduce partial volume effects and potentially lower sensitivity to small focal areas of inflammation. While 2D mapping is the standard approach, 3D myocardial T2 mapping methods have been described that allow whole heart tissue characterization and acquisition of data at isotropic and higher spatial resolution [31, 35]. 3D T2 mapping techniques have been accomplished with free-breathing and self-navigation techniques, utilizing under sampling strategies and compressed sensing reconstruction to accelerate scan times [37, 40].

### K-space sampling

Conventional Cartesian sampling remains the standard approach and sampling trajectories that employ linear and centric view ordering are commonly used for single-shot, 2D T2 mapping. Radial and hybrid cartesian-radial trajectories have also been implemented, primarily in 3D T2 mapping techniques [16, 37, 41]. Radial trajectories can reduce motion sensitivity and afford higher spatial resolution. The under sampled k-space can however result in inherent loss of SNR compared to Cartesian sampling [42].

### Multi-parametric

Investigational techniques have been described that produce T2 maps jointly with other relaxation times by employing multiple magnetization preparation modules in a single image acquisition. This allows co-registered parametric maps to be generated [36, 43–45]. Magnetic resonance fingerprinting based techniques map multiple tissue properties simultaneously by characterizing the signal evolution over time together with dictionary simulation and template matching. T2 maps have been determined simultaneously with T1, proton density, T1ρ and fat fraction [46–48].

### Commercially available 2D T2 mapping methods

**Fig. 4:**
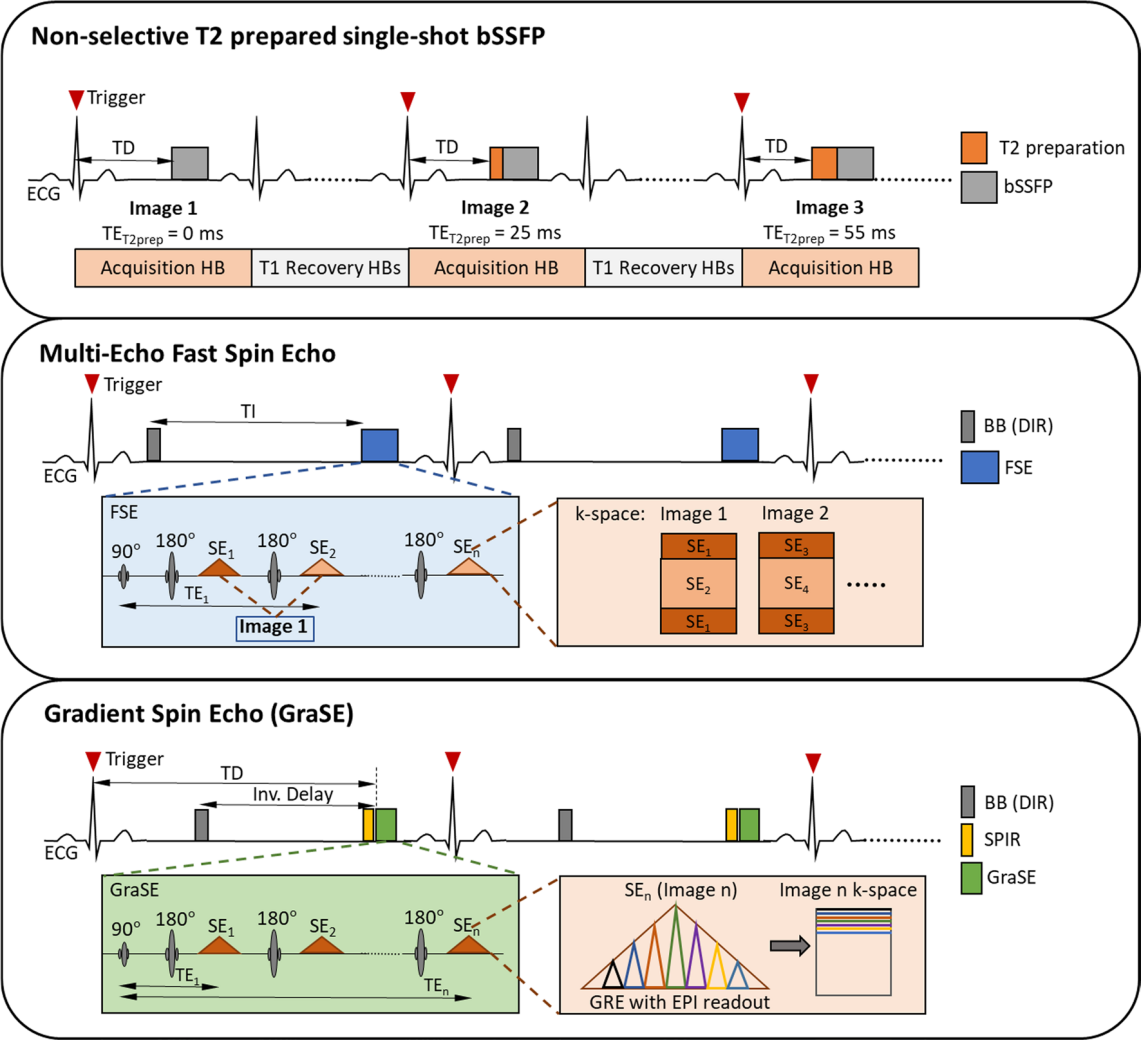
Three Principal Pulse Sequences Used for Myocardial T2 Mapping. While several variations have been described in the literature, only the three techniques shown here are commercially available. ECG—Electrocardiogram, TD—trigger delay, TE—echo time, HB—Heartbeat, bSSFP—balanced steady state free precession, TI—nversion ime, FSE—fast spin echo, SE—spin echo, BB—black blood, DIR—dual inversion recovery, GRE—gradient recalled echo, EPI—echo planar imaging, SPIR—spectral presaturation with inversion recovery

Although a wide range of T2 mapping strategies exist, each having advantages that may best fit the clinical or research demand, currently most CMR centers use one of the following commercially available 2D pulse sequences in their clinical workflow: single-shot T2-prepared bSSFP or T2p-GRE [7], multi-echo fast spin echo (multi-echo FSE) [6, 32], or gradient spin echo (GraSE) [33] (Fig. 4) [49]. Commonly implemented acquisition strategies and technical considerations for these pulse sequences are further summarized below. The Society for Cardiovascular Magnetic Resonance (SCMR) and European Association for Cardiovascular Imaging (EACVI) recommend the use of T2p-bSSFP or T2p-GRE pulse sequences [50].

### Single-shot T2 prepared bSSFP

This mapping technique utilizes a T2 preparation module immediately followed by a single-shot bSSFP readout module [25]. T2 preparation is non-selective and may use composite rectangular or adiabatic 180 $^\circ$ refocusing pulses. Multiple T2-prepared images, usually three, are acquired at different T2 preparation times. The pulse sequence timing delay following the R-wave trigger is adjusted for each image such that each bSSFP readout occurs at the same phase of the cardiac cycle, generally either mid-diastole or end-systole to avoid rapid cardiac motion. Pulse sequence parameters are chosen such that the total acquisition time remains within the duration of a breath hold for most patients. As each image is acquired within one heartbeat for single-shot T2p-bSSFP mapping, it is suggested to adjust the number of recovery heartbeats between source images to approximately three seconds to allow sufficient magnetization recovery.

Specific design parameters such as the number and duration of refocusing pulses in the T2 preparation module could impact the resultant T2 magnetization and therefore the estimated T2 relaxation time. The number of k-space lines acquired between T2 preparation and the center of k-space will affect the influence of the T2 preparation on image contrast, and hence the estimated T2 time. For a T2p-bSSFP pulse sequence, increasing the image field of view and/or resolution in the phase encode direction can reduce T2 weighting, and generally lead to an overestimation of T2 relaxation time. This is exacerbated with linear ordering of k-space. Centric ordering, on the other hand, can cause increased spatial variability and artifacts due to the oscillatory approach to steady state of bSSFP [7]. A GRE readout may be more readily combined with centric ordering, and may be preferred either at 3 T, or in the presence of implanted devices due to the higher sensitivity of bSSFP to off-resonance artifacts.

### Double inversion recovery (DIR) prepared fast spin echo (FSE) or turbo spin echo (TSE)

These are MESE based T2 mapping techniques that generate T2w images with a desired effective echo time (TE) within a breath-hold [6, 32]. These pulse sequences involve a DIR pre-pulse, which consists of a non-selective 180 $^\circ$ IR pulse immediately followed by a selective 180 $^\circ$ inversion recovery (IR) pulse. This effectively nulls the blood signal, leading to black-blood T2w images. Multiple black blood T2w images can be acquired at different TE and then fit pixel-wise to create a T2 map.

### Gradient and spin echo (GraSE)

GraSE-based T2 mapping combines a fast spin echo pulse sequence together with echo-planar imaging (EPI). A train of refocusing 180 $^\circ$ pulses are used to generate a train of spin-echoes, where gradient echoes are sampled before and after each spin echo with an EPI readout. The sampled echoes are used to reconstruct an image with an effective echo time determined by the echo used to encode the central region of k-space. The number of images acquired are determined by the number of 180 $^\circ$ pulses, while the number of gradient echoes surrounding each spin echo is determined by the EPI factor; EPI factors of 3 [8, 18] and 7 [33, 34] have been utilized for myocardial T2 mapping.

GraSE and FSE based T2 mapping are black-blood techniques, which reduce partial volume effects and allow more precise definition of the blood-myocardium border compared to bright-blood T2p-bSSFP. GraSE pulse sequences generally produce significantly longer T2 values than T2p-bSSFP pulse sequences [20]. Imperfect 180° refocusing pulses in fast MESE sequences can cause signal contamination due to the introduction of T1w stimulated and indirect echo pathways. The direct, stimulated, and indirect echoes together contribute to the measured signal intensity of the corresponding echo, resulting in higher-than-expected values. This would prolong signal decay leading to T2 overestimation [51]. Additionally, the longer echo-spacing and EPI readout utilized in GraSE may increase susceptibility to motional blurring and through-plane motion, introducing partial volume effects [17, 34].

## T2 map estimation

As described previously in Eq. 1, a two-parameter model involving two unknowns, ${M}_{0}$ and $T2$, or a three-parameter model having three unknowns ${M}_{0}, T2$, and $C$ are commonly implemented for T2 mapping. A minimum of three points must be sampled along the curve to allow least squares curve fitting of the two-parameter model. Generally, the longest T2prep time is chosen to approximate the expected T2 relaxation time of the myocardium. The number of T2prep source images, T2prep times, and the type of curve-fit are factors that may affect the accuracy and precision of the estimated T2 time [52]. A linear two-parameter fit following logarithmic transformation is frequently used to fit a T2 relaxation time curve when three source images are acquired. The three-parameter model including $C$, facilitates a non-linear fit leading to more accurate T2 time estimates, but also requires the acquisition of additional T2prep images at longer or infinite T2prep times to improve the accuracy and robustness of the fit [41, 52].

## T2 map visualization

Inline T2 maps generated at the scanner are provided with a few pulse sequences and vendors, but it is also possible to generate T2 maps offline from the T2w source images using commercially available CMR post-processing software platforms. The quantitative T2 relaxation times are typically mapped to a color scale. Compared to a gray-scale display, color mapping improves the ability to discern subtle differences in the parameter of interest. The easy visual interpretation can facilitate discrimination of myocardial abnormalities.

The variety of color schemes used to display myocardial T2 maps have also contributed to challenges in visual image interpretation. Rainbow-like color palettes corresponding to colors of the visible spectrum are often the default choice in most post-processing and image visualization toolkits. Although this is useful to highlight small differences in tissue characteristics due to the sharp transitions in color [53], it can also introduce artificial borders if the myocardial T2 relaxation time change corresponds to a color transition but not necessarily a pathological change. In addition, a small myocardial T2 relaxation time change may be obscured if it is within a given color range [54].

Perceptually ordered color scaling (a scale that linearly increases in luminescence) allows easy visual comparison of relative changes in measurement. Heated black-body schemes are available that range from black/cool color at the lower limit to colors that represent increasing warmth to white hot at the upper limit. This scheme has been popular in nuclear medicine applications to present higher signal intensity as warmer colors to the observer. It has also been applied to myocardial T2 mapping where brighter colors represent longer T2 relaxation time. The advantages are easy visibility, which leads the observer to associate an ‘inflamed’ appearance with the red to yellow representation of elevated myocardial T2 relaxation times. Blood in the cardiac chambers is often mapped to an extremely bright/white color because of its four to five-fold longer T2 relaxation time than myocardium. While this large color contrast may facilitate identification of partial volume artifacts, the presence of extremely bright blood adjacent to the myocardium can create challenges in visual interpretation of subtle myocardial T2 relaxation time changes.

A standardized palette with limited hue and luminance levels, specifically designed to accentuate pathological changes in the myocardium corresponding to quantitative T2 time thresholds would be ideal. For a given color scheme, viewing myocardial T2 maps from 3 T at the same window width and center as 1.5 T may visually obscure pathological changes as the dynamic range of T2 times are narrower in the 3 T map. Regardless of the color scale implemented, adjusting the window width and center of the color look up table can provide certain user flexibility to enhance contrast and visibility in areas of interest. An optimal data display is key to ensure accurate visualization of the magnitude and extent of myocardial T2 time prolongation to the user.

## T2 map post-processing

Because numerous factors can affect the T2 relaxation time estimate, including choice of pulse sequence, preparation pulses, field strength, and even the number of acquired k-space lines, it is critical to consider these effects when using any T2 mapping technique. If acquiring a T2 map, at least one should be acquired in the SAx orientation, with the remaining T2 maps determined by the clinical indication [50, 55]. T2 maps are usually acquired at mid or end-diastole to minimize cardiac motion. Acquiring T2 maps at end-systole when the myocardium is thickest can facilitate avoidance of blood pool contamination within the measurement region of interest (ROI) [56], although timing within the cardiac cycle is critical to avoid the rapid myocardial motion of systolic contraction or early diastolic filling. The blood T2 time is much longer than that of myocardium, and inclusion of any blood pool pixels within the ROI can drastically alter the estimated myocardial T2 time. For assessment of diffuse disease and global evaluation, a single septal ROI on a mid-SAx map to avoid artifacts from extra-myocardial structures is recommended [50]. For the evaluation of focal or patchy disease, the ROI may be drawn based on visual impression by an experienced observer and should be at least 20 pixels, and one long axis map should always be acquired [50, 57]. The American Heart Association (AHA) 17-segment model may be used for regional T2 relaxation time quantification, with exclusion of the terminal apical segment which is thin and easily contaminated by blood signal [58].

## Myocardial T2 relaxation times and variability

Exact T2 relaxation times are often reported in studies, but differences in CMR platform, pulse sequences, and post-processing result in significant heterogeneity. Recently Snel et al. performed a meta-analysis of T2 times for various myocardial pathologies [49]. The extant studies on myocardial T2 mapping suggest that T2 times can reliably differentiate between healthy controls and patients with myocardial infarction, dilated cardiomyopathy, myocarditis, and heart transplant [49]. There were insufficient studies on hypertrophic cardiomyopathy, cardiac sarcoidosis, Anderson-Fabry disease, systemic lupus erythematosus, and cardiac amyloidosis to analyze covariates and publication bias, though reported myocardial T2 relaxation times were longer in all except Anderson-Fabry without left ventricular (LV) hypertrophy [49].

Longer T2 relaxation time may be associated with female sex, advancing age, and apical segments [59]. There is significant heterogeneity of reported myocardial T2 relaxation time between studies; field strength, pulse sequence, and CMR vendor account for much of the variation [20]. Interestingly, a recent meta-analysis on healthy participants found no significant impact of age or sex on myocardial T2 time [20]. Hydration status has been found to significantly impact myocardial T2 relaxation time, as might be expected given the relationship between T2 relaxation time and tissue water content [60]. These findings emphasize the importance of maintaining control over scan parameters and patient hydration, as well as generating site specific reference ranges. The SCMR consensus statement on parametric mapping, endorsed by the EACVI, recommends that local reference ranges should be generated using the same scanner, pulse sequence, processing, and analysis as the intended application [50]. Normal myocardial T2 time range is defined as the mean ± 2 standard deviations of the local reference cohort [50].

## Relaxation times and sensitivity to pathology

Cardiovascular disease leads to pathological alterations in the myocardial tissue structure and composition. These changes are associated with a change in myocardial relaxation times. Depending on their sensitivity, myocardial relaxation times may find clinical utility for assessment of specific myocardial diseases. Since T1 and T2 reflect tissue water characteristics, prolonged relaxation time may indicate pathological changes in the state of myocardial water. As native T1 depends on intracellular and extracellular/interstitial factors, myocardial T1 times are used in the assessment of ischemic and nonischemic cardiomyopathy [61] to distinguish focal pathologies such as acute infarct, chronic scar, infiltration, and diffuse fibrosis [62]. Structural changes associated with diffuse fibrosis such as the expansion of the extracellular matrix can be better characterized by T1 mapping in the presence of extracellular contrast agents, allowing one to determine the extracellular volume [63, 64]. Native T1 relaxation time is also sensitive to edema and iron overload [65–67].

T1ρ, the T1 relaxation time in the rotating frame, represents the longitudinal relaxation decay in the presence of an on-resonance spin-lock RF field. Presently, T1ρ methods are experimental and not in clinical use. T1ρ relaxation time is always longer than T2 relaxation time and equals T2 relaxation time in the absence of a spin-lock field [68]. T1ρ relaxation time is sensitive to the interactions between water and macro-molecules like collagen and proteoglycans. Regions of scar or infarcted tissue, which is composed of collagen and fibrous tissue, demonstrate increased T1ρ relaxation times, attributed to increased water mobility [69]. Thus, T1ρ relaxation time has shown sensitivity to edema in acute and chronic myocardial infarction [70]. As myocardial T1ρ relaxation time can be sensitive to ischemic and nonischemic cardiomyopathy without the need for contrast agents, it is a possible alternative to late gadolinium enhancement (LGE) imaging used to detect myocardial scar.

Elevated myocardial T2 relaxation times are used as an index of myocardial edema [7, 71, 72]. Short T2 relaxation time on the other hand, may be indicative of intramyocardial hemorrhage and iron overload, but with less sensitivity than T2* relaxation time [73]. T2 relaxation time is also sensitive to oxygenation changes and has been utilized in myocardial blood-oxygen-level-dependent (BOLD) imaging to perform cardiac stress testing [74]. Myocardial T2* relaxation time is the most sensitive to iron content and is used in the assessment of iron accumulation [75, 76].

The sensitivity of a specific relaxation time to pathological changes may be sufficient to serve as a single marker of myocardial disease or may improve diagnostic confidence when jointly assessed with others. Since the initial use of myocardial T2 mapping to investigate ischemia and reperfusion, it has been clinically and/or experimentally applied to myocardial infarction, cardiac transplant, cardiomyopathies, myocarditis, rheumatologic conditions with cardiac involvement, valvular heart disease, athlete’s heart, muscular dystrophy, chronic kidney disease with cardiac involvement, cardio-oncology and more (Table 1 and Table 2) [71, 77–90]. The following sections focus on cardiovascular disease-specific clinical applications of myocardial T2 mapping.

**Table 1:** Clinical applications of myocardial T2 mapping

Conditions associated with prolonged myocardial T2 relaxation time
Acute coronary syndrome
Myocarditis
Heart transplant rejection
Dilated cardiomyopathy
Amyloidosis
Sarcoidosis
Heart Failure
Hypertrophic cardiomyopathy
Takotsubo cardiomyopathy
Peripartum cardiomyopathy
Rheumatologic disease with cardiac involvement
Anderson-Fabry cardiomyopathy
Cancer therapy related cardiac injury
Neuromuscular diseases
Athlete’s heart
Aortic stenosis

**Table 2:** Advantages of T2 mapping in selected myocardial diseases. The meta-analysis from Snel et al. included sufficient studies to conclude that T2 relaxation time is significantly prolonged above healthy subjects for all diseases included in this table except cardiac amyloidosis. Since the time of its publication, additional studies have shown T2 mapping as an accurate discriminator between AL and transthyretin amyloidosis as well as an accurate prognosticator (see Cardiac Amyloidosis section of main text). The rightmost column adds important advantages of T2 mapping

Disease	Weighted mean T2 relaxation time at 1.5 T	Number of studies in meta-analysis	Clinical utility of T2 mapping
Myocardial infarction	58.5 ± 5.8 ms vs. 49.3 ± 2.6 ms in controls	31	T2 mapping differentiates acute vs. chronic myocardial infarction
			T2 mapping is used in measuring area at risk
			T2 mapping identifies intramyocardial hemorrhage
Heart transplant	54.6 ± 5.2 ms vs. 49.2 ± 2.5 in controls	11	T2 mapping is a reliable surrogate for direct tissue assessment in transplant rejection
			Prolonged T2 time may identify patients that will benefit from immunosuppression modification despite negative endomyocardial biopsy
Myocarditis	61.9 ± 11.5 ms vs. 54.4 ± 5.9 ms in controls	19	Prolonged T2 time corresponds to myocardial edema and inflammation on biopsy
			Abnormal T2 relaxation time has high sensitivity for diagnosing acute myocarditis
			Persistently prolonged T2 relaxation time is associated with increased adverse cardiac events
Amyloidosis	55.3 ± 4.2 ms vs. 50.2 ± 2.7 in controls	2	Local toxicity of amyloid deposits results in longer T2 time, particularly in light chain (AL) amyloidosis
			T2 mapping helps differentiate AL from ATTR amyloidosis
Dilated cardiomyopathy	62.9 ± 5.7 ms vs. 55.4 ± 3.5 in controls	9	T2 mapping improves early detection of dilated cardiomyopathy, prior to left ventricular dysfunction
			Shorter T2 time in patients with successful reverse myocardial remodeling after goal directed medical therapy

Data printed with permission from: Snel GJH, van den Boomen M, Hernandez LM, Nguyen CT, Sosnovik DE, Velthuis BK, Slart RHJA, Borra RJH, Prakken NHJ. Cardiovascular magnetic resonance native T2 and T2* quantitative values for cardiomyopathies and heart transplantations: a systematic review and meta-analysis. Journal of Cardiovascular Magnetic Resonance. 2020;22(1):34. 10.1186/s12968-020-00627-x

## Myocardial ischemia

Early canine studies showed that permanent coronary artery occlusion results in redistribution of water from the interstitial space to the intracellular space likely by failure of transmembrane ATP dependent ion transporters [91]. Ischemia with reperfusion further alters myocardial fluid balance by increasing interstitial water content, resulting in net gain of myocardial water secondary to cellular and vascular injury [91, 92]. T2 mapping, which is both sensitive and specific for myocardial edema, is an ideal imaging modality for characterization of these injured infarct and peri-infarct zones.

Baseline myocardial T2 time for patients with both ST segment elevation myocardial infarction (STEMI), and non-ST segment elevation myocardial infarction (NSTEMI), is significantly longer in the infarct zone, compared with remote zones [93]. T2 relaxation time decreases to near normal in the infarct zone at 6 months after reperfusion and discriminates AMI from chronic infarction with higher diagnostic accuracy than semi-quantitative T2w methods [93]. T2 mapping also outperforms T2w imaging for identification of infarct related artery and estimation of area of myocardial injury in NSTEMI [94]. One factor that may complicate the classification of chronic infarction is intramyocardial hemorrhage (IMH). Work in a canine model of infarction indicates that IMH is associated with persistent edema and cellular inflammation in peri-hemorrhagic territories causing longer T2 time 8 weeks after reperfusion [95]. Therefore, it may be important to consider the presence of hemorrhage when distinguishing infarction chronicity using T2 mapping.

Dynamic changes in myocardial water content occur after reperfusion of infarct territory as shown by a recent porcine study [96]. In ischemia–reperfusion injury, edema peaks at 2 h after reperfusion, normalizes within day 1, then peaks again at day 7 [96]. Moreover, this study showed good correlation of T2 time with myocardial water content—showcasing T2 mapping as a means of dynamically monitoring ischemia–reperfusion injury.

Though controversy over the histopathologic validity of myocardial area at risk (AAR) using T2w imaging exists [97], both T1 and T2 mapping have been used to assess AAR, a key variable in quantification of myocardial salvage [98, 99]. Myocardial salvage index (MSI) is calculated by expressing the difference between AAR and irreversible infarct size (by LGE) as a proportion of AAR [100]. A bimodal edematous myocardial response following reperfusion has also been demonstrated in humans, indicating that assessment of myocardial salvage using T2w imaging should account for timing of the study [100]. However, T2 mapping has shown that AAR, infarct size, and MSI do not vary with time after revascularization [101]. The strengths of T2 mapping over T2w imaging in assessing myocardium at risk are evident in the differing results of the two studies reported above.

T2 mapping is also useful for detection of microvascular obstruction (MVO) and IMH as mentioned above—well-known complications of reperfusion. Both present as a region of shorter T2 time within the infarct core in patients after reperfusion and are associated with increased end diastolic volume (EDV) and decreased LV ejection fraction (LVEF) 6 months after STEMI, emphasizing the role of T2 mapping in prognostication after reperfusion therapy [102]. T2 mapping is highly accurate for the diagnosis of IMH and may be considered as an alternative to T2* mapping [79].

Recently, parametric mapping has been studied as an alternative to gadolinium-based contrast studies for stress testing. Nakamori et al. found that native T1 mapping has better diagnostic accuracy for the detection of myocardial perfusion deficits after exercise than T2 mapping, citing T2 mapping’s sensitivity to field inhomogeneity and detection of confounding myocardial edema after exercise as possible explanations [103]. In healthy study participants, myocardial T2 relaxation time lengthens significantly during adenosine stress testing and the increase is mostly explained by increased extracellular volume (ECV) and increased blood volume [104].

## Myocarditis

Myocarditis is a cause of sudden cardiac death and heart failure [105, 106]. Despite recent technical advances, its diagnosis remains challenging [107]. Endomyocardial biopsy (EMB), a diagnostic gold standard in myocarditis, is an invasive method with limited accuracy due to sampling error arising from the patchy nature of the inflammatory process [108–110]. Given its risks and limitations, biopsy is often not performed in cases of suspected myocarditis. CMR has thus become a key diagnostic tool in myocarditis [107, 111]. Established CMR criteria for the diagnosis of myocarditis are strengthened by parametric mapping [108, 112].

**Fig. 5:**
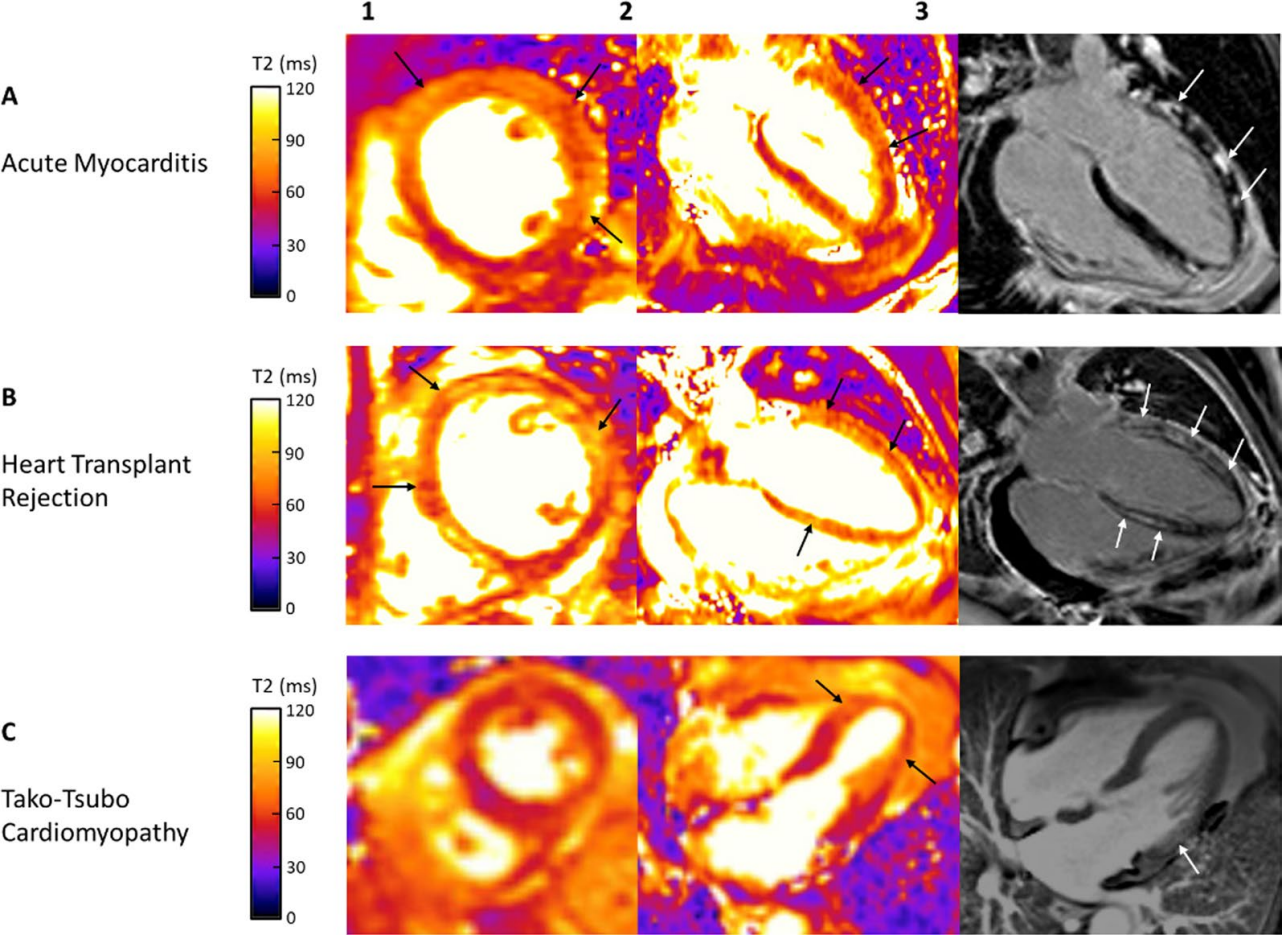
Cardiovascular Magnetic Resonance Imaging in Patients with Presence of Myocardial Inflammation. Panel **1**–**2**: T2 mapping; Panel **3**: late gadolinium enhancement imaging. Panel **1**—midmyocardial short axis view; Panel **2**, **3**—horizontal long axis view. **A** Diffuse myocardial edema/inflammation (Panel **1**–**2**; black arrows) with corresponding prominent midmyocardial to subepicardial nonischemic enhancement (Panel **3**; white arrows) most prominent in the lateral wall in a patient with myocarditis. **B** Patient post heart transplant with diffuse myocardial edema/inflammation (Panel **1**–**2**; black arrows) with striking diffuse linear nonischemic midmyocardial enhancement most prominent in the septal and lateral walls (Panel **3**; white arrows). Findings concerning for heart transplant rejection. **C:** Patient with Takotsubo cardiomyopathy with myocardial edema/inflammation in the mid to apical segments (Panel **1**–**2**; black arrows) with lack of corresponding enhancement in the mid to apical segments. Mild patchy midmyocardial nonischemic fibrosis in the basal lateral wall (Panel **3**; white arrow). Panel **A**, **B**—T2 prepared (rectangular preparation) pulse sequence with balanced steady state free precession (bSSFP) readout utilized to acquire T2 maps during diastole (1.5 T MAGNETOM Avanto, Siemens Healthineers). Panel **C**—T2-prepared bSSFP pulse sequence with the use of rectangular T2 preparation pulse utilized to acquire T2 maps during diastole (1.5 T MAGNETOM Sola, Siemens Healthineers)

Regional and global T2 times are significantly increased in patients with myocarditis, and longer T2 relaxation time correlates with myocardial edema and inflammation in endomyocardial biopsy samples (Fig. 5) [90, 107, 113, 114]. T2 mapping reliably discriminates between biopsy-proven myocarditis and healthy myocardium in patients with recent-onset heart failure presentation [114]. T2 mapping also identifies myocardial involvement in patients with acute chest pain presentation [72]. Diagnostic accuracy of T2 mapping is superior to LGE, global myocardial T1 mapping, and ECV values [114]. This could be partly explained by closer association between free water and myocardial T2 relaxation time, than free water and myocardial T1 [114]. Sensitivity of T2 mapping for acute myocarditis is high irrespective of symptoms, though T2 time specificity is significantly higher in patients with acute chest pain than with heart failure presentation [72, 107, 114]. In patients with chronic symptoms, only T2 mapping among CMR parameters has acceptable diagnostic performance [108].

T2 mapping is useful not only for making the diagnosis, but also for risk stratification [107]. The degree of T2 relaxation time prolongation, and to a lesser extent the percentage of myocardium with prolonged T2 time, are reliable predictors of major adverse cardiac events (MACE) (cardiac death, heart transplantation, ventricular assist device implantation), and heart failure (HF) hospitalization in patients with myocarditis [107]. T2 time tends to shorten after resolution of the inflammation, whereas LGE may persist [107]. Persistent T2 time prolongation after the acute phase correlates with MACE, HF hospitalization, and LV dysfunction making T2 mapping a useful monitoring tool in patients with myocarditis [107].

## COVID-19

Recently, there has been interest in the detection of myocardial involvement in patients with coronavirus disease 2019 (COVID-19). Guidance on CMR during the COVID-19 pandemic can be found in SCMR published recommendations [115–117]. An early study revealed high prevalence of myocardial edema detected by T2 mapping in patients recovered from COVID-19 [118].One third of the patients required hospitalization for COVID-19, and several patients were not completely recovered [118]. A study by the COVIDsortium investigators found a low prevalence of prolonged myocardial T2 time in seropositive healthcare workers 6 months after mild COVID-19 (1 hospitalization out of 74 seropositive cases), and no significant difference in T2 time between seropositive cases and seronegative controls [119].

Research on young athletes recovering from COVID-19 is ongoing. Several studies found prolonged myocardial T2 time in athletes recovered from either mild or asymptomatic COVID-19, while others have not [120–123]. CMR with parametric mapping is selectively recommended for athletes with signs or symptoms of myocardial injury that are recovering from COVID-19 [124]. It is important to consider normal myocardial adaptations to intensive athletic training when evaluating these patients. Larger studies with appropriate controls are needed to elucidate myocardial T2 relaxation time abnormalities associated with COVID-19 in athletes and non-athletes stratified by the severity of disease presentation.

## Heart failure

CMR proves superior diagnostic and prognostic value in HF by delineating specific etiologies, guiding therapeutics, and monitoring response to treatment [125]. T2 mapping may aid in identifying which patients could benefit from immunosuppressive therapies during acute HF [114, 126].

CMR is the most accurate imaging modality for assessment of LV mass, systolic function, pericardium, and myocardial tissue characteristics as they relate to heart failure with preserved ejection fraction (HFpEF) [127, 128]. CMR imaging with cine, tagging, and phase contrast measurements of mitral inflow and mitral annulus velocities can be used to asses LV diastolic function [127]. Patients with HF and moderately reduced LVEF were shown to have longer global LV T2 time than healthy healthy controls, with a similar trend in HFpEF [129]. Global myocardial T2 relaxation time is significantly associated with quality of life, 6 min walking test, glomerular filtration rate and N-terminal pro-brain natriuretic peptide (NT-proBNP) in these patients [129].

Global myocardial T2 relaxation time can serve as an index of compensation in HF, with acute decompensated HF marked by prolonged T2 time, and shortening of T2 time after decongestion [130]. Myocardial tissue may be more susceptible to edema than other striated muscles due to the dynamic nature of venous and lymphatic flow during the cardiac cycle [130].

## Dilated cardiomyopathy

Nonischemic dilated cardiomyopathy (DCM) is a common cause of HF [90, 131]. Myocardial T2 relaxation time is significantly prolonged in patients with chronic presentation of DCM regardless of the degree of LV dysfunction [49, 90, 132]. T2 mapping also improves detection of early DCM when myocardial morphology is difficult to distinguish from athletic myocardial adaptation [133]. T2 time prolongation is not limited to patients with inflammation on endomyocardial biopsy (EMB) [90, 108, 131]. This could be potentially explained by sampling error, presence of edema with number of leukocytes below the required threshold, or microvascular dysfunction causing repetitive ischemia and myocardial edema [90].

A significant percentage of DCM has an underlying inflammatory background, and the presence of inflammatory cells in EMB samples is associated with prolonged myocardial T2 time [90]. In such instances, T2 time prolongation is most prominent in the inferior and lateral walls [90]. T2 mapping could thus be used to better identify patients requiring EMB [90]. No correlation was found between T2 time and presence of viral genomes in EMB samples or grade of fibrosis [90].

Shorter myocardial T2 relaxation time may be used to predict which patients will experience LV reverse remodeling during treatment for DCM, and the reverse remodeling is associated with normalization of T2 time [134]. Therefore, T2 mapping is an important supportive tool for diagnosis, stratification, and monitoring in DCM.

## Hypertrophic cardiomyopathy

**Fig. 6:**
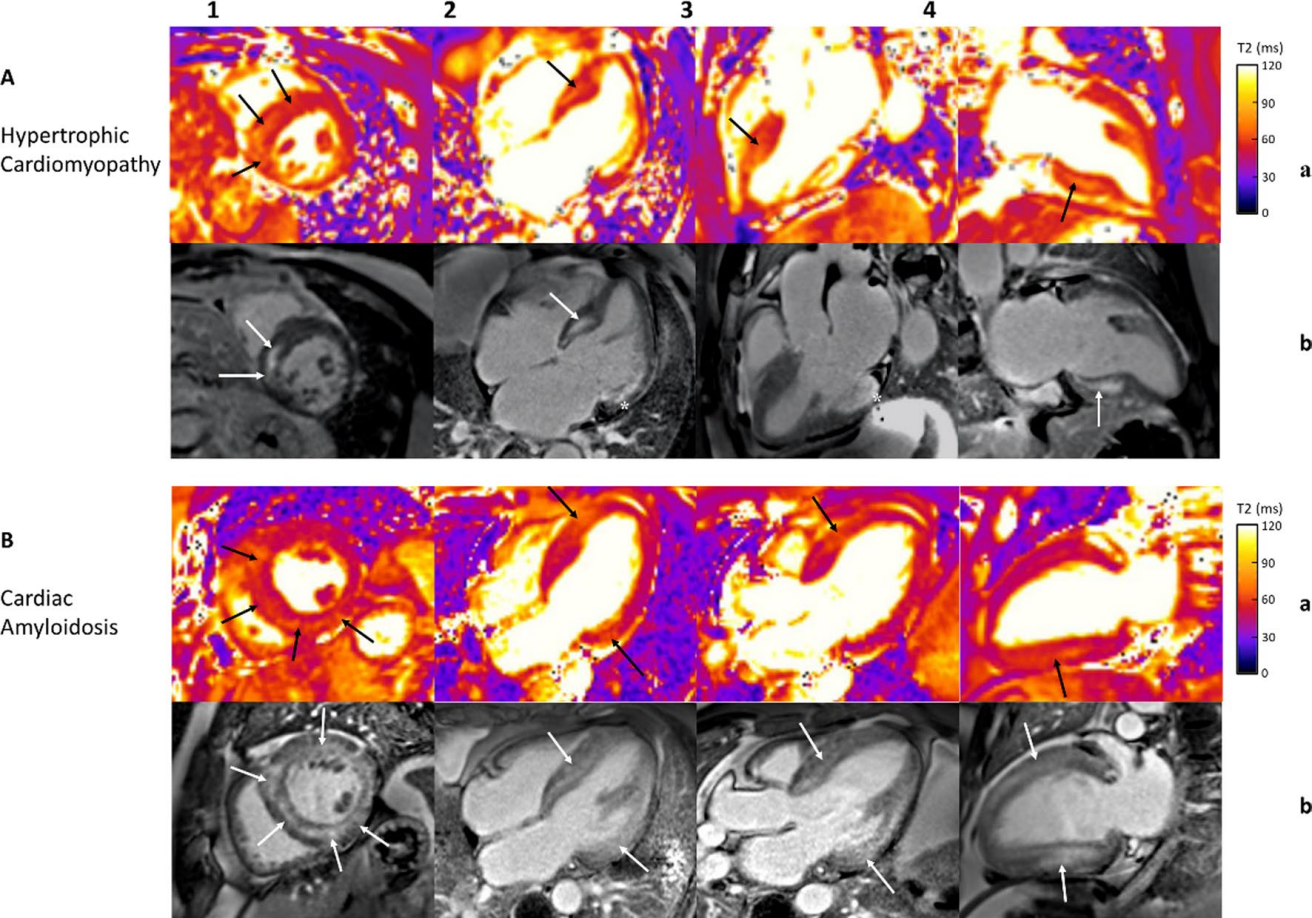
Cardiovascular Magnetic Resonance Imaging in Patients with Increased Left Ventricular Wall Thickness. Panel **a**: T2 mapping; Panel **b**: late gadolinium enhancement imaging. Panel **1**—midmyocardial short axis view; **2**—horizontal long axis view; **3**—three chamber long axis view; **4**—vertical long axis view. **A** Patient with hypertrophic cardiomyopathy with myocardial edema/inflammation in the septal and inferior walls (Panel **a1**–**a4**; black arrows) and prominent midmyocardial nonischemic fibrosis in the basal to mid septal and inferior walls (Panel **b1**–**b2**, **b4**; white arrows). In addition, there is infarct scar along the basal to mid lateral walls (Panel **b2**–**b3**; asterisks). **B** Patient with cardiac amyloidosis with myocardial edema/inflammation in the septal, inferior and lateral walls (Panel **a1**–**a4**; black arrows) with diffuse late gadolinium enhancement throughout the left ventricle (Panel **b1**–**b4**; white arrows). T2-prepared bSSFP pulse sequence with the use of rectangular T2 preparation pulse utilized to acquire T2 maps during diastole (1.5 T MAGNETOM Sola, Siemens Healthineers)

Hypertrophic cardiomyopathy (HCM) is a common cause of sudden cardiac death [135]. CMR is the key tool for its diagnosis and risk stratification with the majority of prognostic assessment attributed to LGE [135]. T2 and T1 relaxation times are significantly prolonged in the non-hypertrophic myocardial segments despite their preserved contractility [135]. This suggests that in HCM, tissue remodeling precedes functional remodeling and parametric mapping can be helpful in making the diagnosis at an early stage of the disease [135]. The degree of T2 time prolongation increases with increasing severity of hypertrophy, and that association is more pronounced than for T1 time prolongation (Fig. 6) [135]. Myocardial edema, and hence T2 relaxation time, might be influenced by collagen accumulation, ischemia, or microvascular dysfunction induced by myocardial hypertrophy and capillary endothelial dysfunction causing increased free water [9, 135].

T2 mapping may aid in the discrimination of LV hypertrophy (LVH) due to strength training vs. HCM [78]. Patients with HCM have significantly longer global LV T2 time than weight lifters with LVH [78].

A recent study in over 150 patients with HCM found that mid-septal T2 relaxation time is shorter than healthy controls [136]. However, another study with similar sample size found prolonged myocardial T2 relaxation time in patients with HCM and an association between T2 time and non-sustained ventricular tachycardia [137]. The findings suggest that prolonged T2 relaxation time could be used as a marker for arrhythmogenicity [137].

## Cardiac transplant

The number of cardiac transplants has increased from less than 500 per year worldwide in the early 1980’s to nearly 6000 in the year 2017 [138]. Over 10% of patients will experience transplant rejection within one year of transplant [139]. Therefore, routine rejection surveillance with EMB is performed frequently throughout the first year after transplant. The complication rate of EMB is low, though improving immunosuppressive regimes have also lowered the diagnostic yield of these procedures which are limited to sampling of endocardial based septal tissue [140]. This increasingly neutral risk–benefit ratio may be augmented by noninvasive tissue characterization with CMR and its accurate morphologic, and parametric analyses.

Both animal and human studies have shown that myocardial T2 relaxation time can be a valuable surrogate for direct tissue assessment in cardiac transplant rejection (Fig. 5). T2 time measurement using a spin echo pulse sequence was positively correlated with degree of histologic rejection in canines that undergo heterotopic cardiac transplant [141]. The same study showed that transplanted hearts have greater myocardial water content than the native heart, and that the increase in water content has a direct linear relationship with myocardial T2 relaxation time [141].

A 2009 review by Butler et al. summarized the early evidence of longer T2 time in rejecting vs non-rejecting human and animal cardiac transplant subjects [142]. Prior to the implementation of contemporary T2 mapping pulse sequences, human studies demonstrated that T2 time is significantly longer in cardiac transplant patients with rejection and that myocardial T2 relaxation time is significantly correlated with histologic grade of rejection [143–145].

More recently, a pilot study by Usman et al. in 53 patients after cardiac transplant, found that T2 mapping accurately diagnosed transplant rejection compared with EMB [146]. Importantly, there were two patients in this study that presented with clinical rejection (no biopsy evidence of rejection, but clinical improvement after immunosuppressive therapy) who had longer myocardial T2 time comparable to biopsy proven rejection patients. Thus, T2 mapping has the potential to identify patients that will benefit from increased immunosuppression despite negative EMB.

A subsequent study by Miller et al. including 22 patients that underwent cardiac transplant found no T2 time prolongation in patients with significant histologic rejection compared with mild or absent histologic rejection [147]. The intervals between transplant and scan were significantly longer in the Usman study, and the study did not statistically adjust for repeated measures in the same subjects over time [146, 147]. These differences in study design and statistical analysis, along with inherent sampling error of EMB, and low numbers of histologic rejection likely account for the discrepant results of the Usman and Miller studies.

Vermes et al. (2018) conducted a similar study in 20 cardiac transplant patients which showed that prolonged basal T2 time diagnosed EMB-positive rejection with 71% sensitivity and 96% specificity using a center specific cutoff [148]. Basal myocardial T2 time was then combined with ECV mapping for a multi-parametric workflow which had the capacity to eliminate 63% of EMB which were negative by CMR criteria and EMB. A multi-parametric approach with age at CMR, global T2 time, and global ECV can diagnose patients with biopsy proven acute rejection with high accuracy [82].

## Amyloidosis

Cardiac amyloidosis, characterized by interstitial amyloid infiltration, leads to myocardial wall thickening and progressive heart failure [84, 149, 150]. Elevated native T1 time and ECV, along with subendocardial or transmural LGE, are highly specific CMR findings [50, 149, 150]. Amyloid deposition and its toxic influence on cardiomyocytes are the most likely cause of myocardial edema seen in EMB and hence prolonged T2 relaxation time (Fig. 6) [84]. While T2 times in amyloidosis are lower than typically reported in myocarditis or myocardial infarction, this may be explained by the fact that active inflammation is rarely present on histology [84]. No correlation was found between visually assessed percent of edema on EMB and myocardial T2 time, though histologic assessment of edema may not always provide reliable information [9, 84].

Differentiation between transthyretin (ATTR) and light chain (AL) amyloidosis is crucial for proper treatment implementation [149]. Despite better prognosis, LV mass tends to be higher, LVEF lower, LGE more prominent, and ECV higher in ATTR amyloidosis [84, 149, 150]. However, myocardial T2 relaxation time is more significantly elevated in AL amyloidosis, with T2 time being a discriminator between ATTR and AL amyloidosis [149]. A novel CMR model for distinguishing AL from ATTR amyloidosis with high accuracy incorporates mean T2 time, age, and right ventricular ejection fraction [151].

Prolonged myocardial T2 time is also an independent predictor of mortality in AL amyloidosis that remains significant after adjustment for ECV and serum biomarkers [84, 150]. Treatment of AL amyloidosis is associated with shortening of myocardial T2 relaxation time [84]. There is no correlation between T2 time and prognosis in patients with ATTR amyloidosis [84, 150].

## Sarcoidosis

**Fig. 7:**
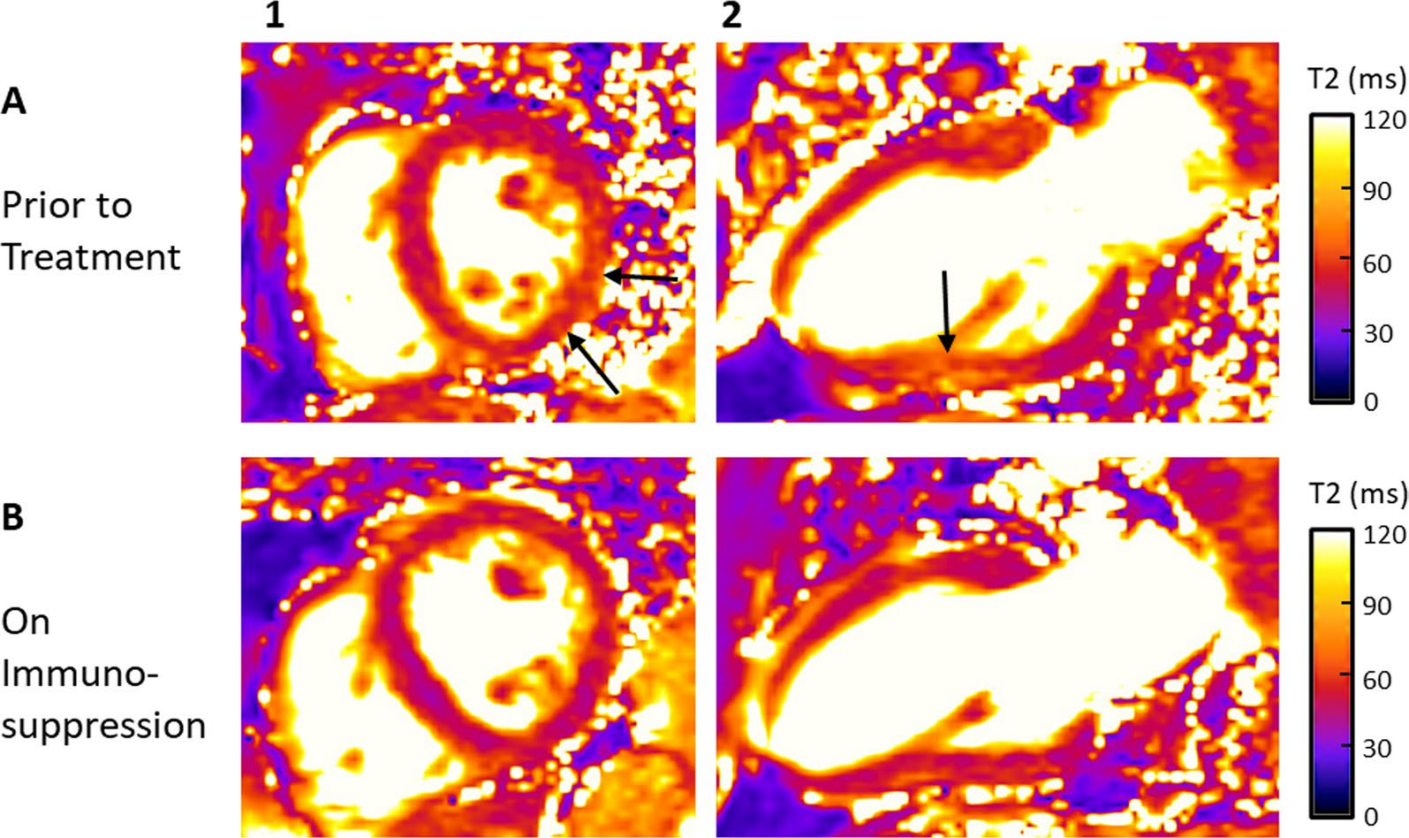
Cardiovascular Magnetic Resonance Imaging in a Patient with Cardiac Sarcoidosis Prior to Treatment (Panel **A**) and on Immunosuppression (Panel **B**). Panel **1**—midmyocardial short axis view; Panel **2**—vertical long axis view. Diffuse myocardial edema/inflammation most prominent in the inferior, and lateral walls (Panel **A**; black arrows). There was interval improvement with no residual myocardial edema/inflammation while on immunosuppressive therapy (Panel **B**). T2 prepared (rectangular preparation) pulse sequence with bSSFP) readout utilized to acquire T2 maps during diastole (1.5 T MAGNETOM Avanto, Siemens Healthineers)

Sarcoidosis is a chronic inflammatory disease of unknown etiology characterized by noncaseating granulomatous tissue infiltration [113, 152]. Cardiac involvement is common and reported in approximately 25% of patients with systemic sarcoidosis [153, 154]. Identification of cardiac sarcoidosis is important due to increased risk of HF and sudden cardiac death [154]. EMB has low sensitivity due to sampling error making the diagnosis challenging [154]. T2 time is prolonged in patients with active cardiac sarcoidosis and is a good discriminator between healthy and inflamed myocardium irrespective of symptoms or disease duration [113, 154]. Prolonged T2 time is associated with ECG abnormalities in patients with sarcoidosis [113, 155–157]. T2 mapping can be used as a supportive tool in diagnosing early cardiac sarcoidosis along with wall motion abnormalities in a non-coronary distribution, and multifocal LGE at more advanced stages of the disease [113, 154]. Significant reductions in myocardial T2 relaxation time and ECG abnormalities occur after anti-inflammatory treatment, thus T2 mapping can be used to monitor treatment efficacy (Fig. 7) [113, 158].

A recent study by Flamée et al. found that only presence of LGE was significantly associated with hard MACE in systemic sarcoidosis, though not all patients underwent T2 mapping [80]. Taken together, these studies indicate a clear role for T2 mapping in the identification of subclinical myocardial inflammation which may herald clinical deterioration, as well as following response to immunosuppressive therapies.

## Cardiac iron overload

Iron overload may occur as a result of hemochromatosis [159], or frequent blood transfusions [50]. The resultant cardiac iron overload leads to HF and lethal arrhythmias [50]. T2* mapping is highly specific and enables quantitative assessment of cardiac and hepatic iron content [159, 160]. It is used in making the diagnosis and monitoring therapy [49]. There is strong correlation between myocardial T2 and T2* times with significantly shorter myocardial T2 time in patients with cardiac iron overload [161, 162]. Patients with thalassemia and chronic iron overload based on T2* mapping have significantly shorter myocardial T2 time than patients without chronic iron overload [87, 163]. Meta-analysis results of T2 mapping in cardiac iron overload patients vs. controls by Snel et al. indicate no significant difference [49]. This is most likely due to limited studies on T2 mapping in cardiac iron overload patients, and that the study by Kritsaneepaiboon et al. had only 3 patients that met criteria for mild cardiac iron overload [49, 164]. More studies are needed to evaluate the role of T2 mapping in cardiac iron overload.

## Anderson-Fabry disease

Anderson-Fabry disease is a rare X-linked recessive lysosomal storage disorder [50, 165–168]. Concentric LVH and eventually HF, is a leading cause of death in this disease [50, 165–168]. CMR is the only imaging modality that discriminates Anderson-Fabry disease from other forms of LVH [167]. Decrease in global T1 time and midmyocardial LGE in the basal inferolateral wall, which is often thin, is associated with focal T1 and T2 time prolongation in the LGE positive areas [50, 165, 168]. T2 relaxation time in LGE positive segments is significantly prolonged in Anderson-Fabry disease compared with other cardiomyopathies [168]. Furthermore, prolonged T2 time in the basal inferolateral wall is a reliable predictor of serum cardiac troponin level in patients with Anderson-Fabry disease [165, 168]. These findings suggest that T2 mapping can be used to monitor myocardial injury during treatment of patients with Anderson-Fabry disease.

## Rheumatologic diseases with cardiac involvement

The autoimmune rheumatologic diseases including systemic lupus erythematosus (SLE), systemic sclerosis, anti-neutrophilic cytoplasmic antibody (ANCA) associated vasculitis, rheumatoid arthritis, and idiopathic inflammatory myositis frequently involve the pericardium and myocardium. T2 mapping may be a preferred non-contrast imaging modality for patients with early and/or reversible myocardial injury. For patients with multi-system disease, and severe renal involvement, gadolinium-based contrast agents should be used with caution [169]—though a recent meta-analysis indicates very low risk of nephrogenic systemic fibrosis in patients with advanced chronic kidney disease with the use of macrocyclic contrast agents [170].

SLE causes myocardial edema that can be detected with T2 mapping even at low levels of SLE disease activity and preserved myocardial function [88, 171]. Patients with active SLE often meet CMR diagnostic criteria for myocarditis despite low symptom burden and infrequent LGE [172]. Even patients with low disease activity have significantly prolonged myocardial T2 time compared with healthy controls and significantly shorter myocardial T2 time after treatment intensification [173]. T2 time prolongation in SLE patients without myocardial symptoms is also a strong predictor of cardiac troponin release [174]. T2 time prolongation may also serve as an index of arrhythmogenicity in patients with SLE and other autoimmune rheumatic diseases [175].

Like SLE and sarcoidosis, systemic sclerosis (SSc) can cause overt or subclinical myocardial injury. T1 mapping is of particular interest in the evaluation of SSc, as it is closely associated with myocardial fibrosis [176]. SSc patients with elevated T1 relaxation time have significantly longer myocardial T2 relaxation time than those without elevated T1 time [177]. Many patients with SSc have prolonged myocardial T2 time without LGE, and the degree of prolongation may be even greater than patients with SLE [88]. SSc patients with recent cardiac signs or symptoms have longer T2 time than those without, and prolonged T2 time may predict future adverse cardiac events [178]. More studies are needed to confirm that T2 time can be used as an accurate cardiac prognosticator in SSc [77].

Idiopathic inflammatory myopathies (IIM) are also associated with myocardial T2 relaxation time prolongation. Several studies found significant prolongation of myocardial T2 relaxation time in a heterogeneous group of patients with IIM, including polymyositis, dermatomyositis, anti-synthetase syndrome, necrotizing autoimmune myopathy, inclusion body myositis, and overlap syndromes [179–181]. Myocardial T2 time can be elevated even with normal LVEF and no LGE in these patients [179, 181]. It may be difficult to distinguish patients with IIM and cardiac involvement from patients with acute viral myocarditis if the clinical history is unclear. Myocardial T2 relaxation time is elevated in both conditions and can distinguish patients from controls, but has not been found to differentiate acute viral myocarditis from IIM with cardiac involvement [180]. Patients with IIM undergoing treatment are noted to have shorter myocardial T2 time that parallels improvement in serum inflammatory markers [181].

Patients with rheumatoid arthritis have significantly longer myocardial T2 time compared with healthy controls with no difference in T2 time between LGE positive and negative rheumatoid arthritis patients [182]. Myocardial T2 relaxation time is positively correlated with disease duration and has been found to be the best CMR parameter for discriminating patients with rheumatoid arthritis from healthy controls [182].

ANCA associated vasculitis may present with subclinical myocardial inflammation and prolonged myocardial T2 relaxation time, while nearly half of patients are LGE negative [183]. It is not yet known whether myocardial T2 time is a meaningful prognosticator in these patients.

These studies highlight the clinical range of T2 mapping and the many avenues for exploration of T2 mapping as a predictor of outcomes. A key strength of T2 mapping in the context of these autoimmune inflammatory disorders is in the identification of reversible myocardial irregularities prior to clinical deterioration, and its use as a valuable surveillance tool and treatment guide.

## Aortic stenosis

Aortic stenosis (AS) is associated with progressive myocardial remodeling with LVH, supply–demand ischemia, cardiomyocyte death, and accumulation of interstitial collagen contributing to HF [184, 185]. As the degree of biopsy proven myocardial fibrosis increases, so do native T1 time and ECV fraction [186]. Recent work has shown that ECV is most accurate for quantifying myocardial fibrosis in the absence of significant myocardial inflammation [187]. Thus, as a reliable index of myocardial inflammation, T2 mapping adds valuable information to the interpretation of ECV mapping in myocardial pathology. In severe AS, global myocardial T2 relaxation time is significantly elevated, exhibiting little overlap with healthy controls [85]. In addition, global myocardial T2 time shows a positive linear association with LV mass index in patients with severe AS [85]. Transcatheter aortic valve replacement (TAVR) results in decreased global myocardial T2 time, commensurate with decreased LV mass index over 6 month follow-up [188]. Patients with the longest pre-TAVR T2 time show the greatest reduction in LV EDV and more improvement in LVEF at 6 months post-TAVR. Myocardial T2 relaxation time also shows a positive linear relationship with aortic valve pressure gradient in severe AS [189]. Taken together, these studies indicate that T2 mapping adds important information about the extent of cardiac decompensation in severe AS, independent of myocardial fibrosis. Detection of myocardial edema in severe AS may also aid in prediction of reverse remodeling after TAVR.

## Neuromuscular disorders

Cardiac involvement in patients with neuromuscular disorders such as Duchenne muscular dystrophy (DMD), Becker muscular dystrophy, Emery-Dreifuss muscular dystrophy, limb-girdle muscular dystrophies, myotonic dystrophies, and others is common [190].

Patients with DMD have significant cardiomyocyte necrosis, interstitial fibrosis, fatty infiltration, and interstitial edema which may lead to HF and arrhythmias [190–192]. Early work using T2 mapping in DMD showed that patients have higher heterogeneity of T2 relaxation time across the LV compared to controls [193]. A study in 12 DMD patients found significantly shortened T2 time in the anteroseptal segment compared with healthy controls [194]. Myocardial T2 time in patients with myotonic dystrophy type 2 was significantly prolonged in basal, mid, and apical maps [195]. However, prolonged T2 relaxation time has not been found in patients with facioscapulohumeral muscular dystrophy [83]. More studies are needed to characterize myocardial T2 relaxation time in these rare diseases.

## Athletes

The athlete’s heart may undergo adaptive remodeling that bears superficial resemblance to pathological remodeling in cardiac disease states [196]. Septal myocardial T2 time is significantly elevated in early idiopathic DCM patients compared with athletes and may help to differentiate these similar myocardial morphologies [133].

Studies involving elite breath-hold divers and male triathletes did not find significant T2 time prolongation compared to controls [197, 198]. However, a study of 30 ultra-marathon runners found significantly longer myocardial T2 time (chronically) compared with healthy controls despite no significant differences in CMR markers of myocardial fibrosis [199]. Recently, T2 mapping was shown to accurately discriminate strength-trained athletes with LVH from patients with HCM [78]. Prolonged myocardial T2 relaxation time has also been found in athletes with ventricular rhythm disturbances compared with control athletes [200]. These studies suggest that T2 mapping adds important data to the analysis of athlete’s heart and prolonged T2 time may indicate myocardial pathology.

## Takotsubo cardiomyopathy

Takotsubo cardiomyopathy (TTCM) is characterized by reversible mid and apical ventricular inflammation and wall motion abnormalities [201–203]. T2 mapping detects significant edema in mid and apical LV segments of Takotsubo patients compared with basal (remote) segments and healthy controls (Fig. 5) [72]. The basal to apical gradient of increasing myocardial T2 relaxation time in TTCM was confirmed by Aikawa et al. with shortening of T2 time toward normal values over 3 months follow-up [204]. Recent work has shown that T2 time in Takotsubo patients is also higher in myocardial regions with normal wall motion compared with controls [205]. A combination of T2 mapping and native T1 mapping can accurately diagnose TTCM [206].

## Peripartum cardiomyopathy

Peripartum cardiomyopathy (PPCM) is a poorly understood condition associated with decreased LV systolic function [207]. A recent study in 40 patients with PPCM found a very low prevalence of LGE and detected no edema by T2w imaging [208]. Only one study using T2 mapping in PPCM patients was identified; it reported significantly prolonged T2 relaxation time in PPCM patients compared to healthy controls [209]. Persistence of LV dysfunction after 6 months follow-up can be predicted by longer myocardial T2 time at baseline [209]. T2 mapping may be used as a prognosticator for patients with PPCM, but more studies are needed to validate these recent findings.

## Chronic kidney disease

Cardiovascular diseases are a leading cause of excess morbidity and mortality in patients with chronic kidney disease (CKD) and end stage renal disease (ESRD) [210]. Shortened myocardial T2 and native T1 relaxation times parallel reductions in LV mass index in patients with ESRD after undergoing hemodialysis [211]. This finding is consistent with recent work which indicates that whole body fluid status may significantly impact myocardial T2 time [60]. Similarly, patients with advanced CKD and LVH have significantly longer myocardial T2 time than patients with HCM or hypertensive cardiomyopathy [136]. Native T1 relaxation time is elevated in patients with CKD, and is highly correlated with myocardial T2 time, indicating dual impact of myocardial fibrosis and edema in LV remodeling [136].

## Cancer therapy-related cardiac injury

Many cancer therapeutics such as the anthracyclines, HER2/neu inhibitors, tyrosine kinase inhibitors, proteasome inhibitors, immunotherapies, and radiation may cause myocardial injury, leading to dysfunction and morbidity [212]. Studies using T2 mapping have consistently found prolonged T2 relaxation time indicating myocardial edema after treatment with anthracycline drugs [11, 213–215]. A porcine study found that prolonged myocardial T2 time was the earliest marker of reversible myocardial toxicity in anthracycline treated pigs [11]. Furthermore, this animal study correlates myocardial T2 time with the histopathologic finding of intramyocardial vacuolization [11]. Other studies have investigated patients undergoing treatment with anthracycline drugs and HER2/neu inhibitor therapy in series finding that this combination is also associated with myocardial edema [81, 216].

A growing arsenal of targeted immunotherapies for cancer creates many opportunities to use T2 mapping in the study of their adverse myocardial effects. Immunotherapeutic agents such as chimeric antigen receptor T-cell therapy, and immune checkpoint inhibitors are also associated with cardiac dysfunction [217, 218]. In patients undergoing CMR, T2 mapping could serve as a valuable index of early, reversible myocardial injury attributed to novel antineoplastic drugs.

## T2 mapping in the pediatric population

Contemporary studies using parametric mapping in the pediatric population primarily utilize T1 and/or ECV mapping [219]. T2 mapping in the pediatric population has predominantly been utilized in patients with myocarditis and heart transplant recipients. Systematic studies of CMR mapping in pediatric patients are scarce since mapping is not routinely performed in many pediatric academic institutions [220]. There is also a scarcity of studies investigating normal ranges of T2 time in healthy pediatric patients [221]. Additionally, T2 mapping may pose more challenges in the pediatric population due to high heart rate and heart rate variability, motion artifact, and need for sedation prior to CMR in the youngest subjects [220]. Breath-hold compliance and difficulty remaining still during the exam may be challenges experienced with non-sedated children. Smaller heart size may also necessitate the acquisition of T2 maps at higher spatial resolution than adult maps. In a study by Alsaied in 102 healthy pediatric subjects, T2 relaxation time at 1.5 T demonstrated a non-significant negative correlation with age, and no correlation with heart rate [222]. There were no differences in T2 time between genders [222]. In another study performed at 3 T that included 38 healthy children, there were no significant correlations between T2 time and age, height, weight, or body surface area [221]. However, significantly longer T2 relaxation times were observed in females compared with males, and in the pubertal (ages 13–15 years) compared with prepubertal periods (ages 9–12 years) [221]. The lack of influence of age on the T2 relaxation time could derive from the fact that only a small age range was analyzed [221]. The generation of a site-specific normal T2 time range is imperative for the pediatric application of T2 mapping.

Diagnosis of myocarditis in the pediatric population is challenging because of the wide spectrum of clinical presentation and heterogenous clinical course [223]. Similar to the adult population, T2 times were found to be significantly elevated in pediatric patients with clinically suspected acute myocarditis, with longer relaxation time in patients with reduced LVEF [220, 223]. Cornicelli et al. reported T2 time prolongation in 90% of acute myocarditis cases missed by original Lake Louise criteria, emphasizing the difference in sensitivity of the original and updated Lake Louise criteria for the diagnosis of myocarditis [220, 223]. Interestingly, Isaak et al. demonstrated comparable diagnostic accuracy between non-contrast scoring based on quantitative mapping parameters and the updated Lake Louise criteria [223]. Since the T2 relaxation time was shorter in myocarditis patients under the age of 18 years than in patients ages 18–21 years, characterization of site and age specific normal T2 time range is warranted for the delineation of cutoff values [223].

Wang et al. conducted a study on 31 patients with clinically suspected myocarditis and no evidence of focal myocardial edema or necrosis/fibrosis visible on conventional CMR at 3 T [224]. The study did not demonstrate any significant difference between T2 time in the myocarditis and control groups [224]. Inclusion of patients with relatively mild symptoms, small sample size, varying and predominantly long intervals from symptom onset to CMR study, and lack of histologic validation are limitations of this study [224].

While CMR with quantitative T2 mapping has been extensively studied as a screening method for acute allograft rejection in adult heart transplant patients, there are limited data on its application in the pediatric population [225]. Sethi et al. found a significant difference between both mean and maximum T2 relaxation time in a rejection group versus the non-rejection group [225]. Similar to prior reports in the adult population, prolongation of T2 relaxation time is observed in higher EMB grades [143, 225]. In a study conducted by Husain in pediatric heart transplant patients, prolonged T2 time correlated with reduced systolic function as assessed by LVEF, global circumferential strain and global longitudinal strain which may indicate disease progression [226]. There was a weak correlation between higher rejection score and T2 relaxation time [226]. The presence of even low-grade coronary allograft vasculopathy was associated with longer maximum segmental T2 time [226].

## Conclusions

The clinical applications of T2 mapping are widespread and impactful. Technology allowing rapid, high-quality acquisition of myocardial T2 maps in a variety of disease states is steadily advancing. Myocardial T2 maps add specific, complementary information about myocardial edema to other parametric CMR studies such as ECV and native T1 mapping. This information can be used for diagnosis, risk stratification, and guiding or monitoring treatment of myocardial disease.

## Data Availability

Review article, not applicable.

## Abbreviations

AAR: Area at risk
AHA: American Heart Association
AL: Light chain amyloidosis
AMI: Acute myocardial infarction
ANCA: Anti-neutrophilic cytoplasmic antibody
AS: Aortic stenosis
ATP: Adenosine triphosphate
ATTR: Transthyretin amyloidosis
BOLD: Blood-oxygen level dependent
bSSFP: Balanced steady state free precession
CKD: Chronic kidney disease
CMR: Cardiovascular magnetic resonance
COVID-19: Coronovirus diseases 2019
DCM: Dilated cardiomyopathy
DIR: Double inversion recovery
DMD: Duchenne muscular dystrophy
EACVI: European Association of Cardiovascular Imaging
ECG: Electrocardiogram
ECV: Extracellular volume
EDV: End diastolic volume
EMB: Endomyocardial biopsy
EPI: Echo planar imaging
ESRD: End stage renal disease
FLASH: Fast low angle shot
FSE: Fast spin echo
GFR: Glomerular filtration rate
GraSE: Gradient spin echo
GRE: Gradient recalled echo
HCM: Hypertrophic cardiomyopathy
HER2: Human epidermal growth factor 2
HF: Heart failure
HFpEF: Heart failure with preserved ejection fraction
HLA: Horizontal long axis
IIM: Idiopathic inflammatory myopathy
IMH: Intramyocardial hemorrhage
LGE: Late gadolinium enhancement
LV: Left ventricle/left ventricular
LVEF: Left ventricular ejection fraction
LVH: Left ventricular hypertrophy
MACE: Major adverse cardiac event
MESE: Multi-echo spin echo
MSI: Myocardial salvage index
MVO: Microvascular obstruction
NSTEMI: Non-ST segment elevation myocardial infarction
NT-proBNP: N-terminal pro-brain natriuretic peptide
PPCM: Peripartum cardiomyopathy
ROI: Region of interest
SAx: Short axis
SCMR: Society for Cardiovascular Magnetic Resonance
SLE: Systemic lupus erythematosus
SNR: Signal-to-noise ratio
SSc: Systemic sclerosis
STEMI: ST segment elevation myocardial infarction
STIR: Short tau inversion-recovery
T1w: T1 weighted
T2prep: T2 prepared
T2w: T2 weighted
TAVR: Transcatheter aortic valve replacement
TE: Echo time
TSE: Turbo spin echo
TTCM: Takotsubo cardiomyopathy
VLA: Vertical long axis

## References

[1] Brown JJ, Peterson TM, Slutsky RA (1985). Regional myocardial blood flow, edema formation, and magnetic relaxation times during acute myocardial ischemia in the canine. Invest Radiol.

[2] Higgins CB, Herfkens R, Lipton MJ, Sievers R, Sheldon P, Kaufman L (1983). Nuclear magnetic resonance imaging of acute myocardial infarction in dogs: alterations in magnetic relaxation times. Am J Cardiol.

[3] Johnston DL, Liu P, Rosen BR, Levine RA, Beaulieu PA, Brady TJ (1987). In vivo detection of reperfused myocardium by nuclear magnetic resonance imaging. J Am Coll Cardiol.

[4] Bouchard A, Reeves RC, Cranney G, Bishop SP, Pohost GM (1989). Assessment of myocardial infarct size by means of T2-weighted 1H nuclear magnetic resonance imaging. Am Heart J.

[5] Johnston DL, Mulvagh SL, Cashion RW, O’Neill PG, Roberts R, Rokey R (1989). Nuclear magnetic resonance imaging of acute myocardial infarction within 24 hours of chest pain onset. Am J Cardiol.

[6] Simonetti OP, Finn JP, White RD, Laub G, Henry DA (1996). “Black blood” T2-weighted inversion-recovery MR imaging of the heart. Radiology.

[7] Giri S, Chung Y-C, Merchant A, Mihai G, Rajagopalan S, Raman SV (2009). T2 quantification for improved detection of myocardial edema. J Cardiovasc Magn Reson.

[8] Fernández-Jiménez R, Sánchez-González J, Aguero J, Del Trigo M, Galán-Arriola C, Fuster V (2015). Fast T2 gradient-spin-echo (T2-GraSE) mapping for myocardial edema quantification: first in vivo validation in a porcine model of ischemia/reperfusion. J Cardiovasc Magn Reson.

[9] Friedrich MG (2010). Myocardial edema—a new clinical entity?. Nat Rev Cardiol.

[10] Abdel-Aty H, Simonetti O, Friedrich MG (2007). T2-weighted cardiovascular magnetic resonance imaging. J Magn Reson Imaging JMRI.

[11] Galán-Arriola C, Lobo M, Vílchez-Tschischke JP, López GJ, de Molina-Iracheta A, Pérez-Martínez C (2019). Serial magnetic resonance imaging to identify early stages of anthracycline-induced cardiotoxicity. J Am Coll Cardiol.

[12] Takeuchi M, Sekino M, Iriguchi N, Ueno S (2004). Dependence of the spin-spin relaxation time of water in collagen gels on collagen fiber directions. Magn Reson Med Sci MRMS.

[13] Scholz TD, Fleagle SR, Burns TL, Skorton DJ (1989). Nuclear magnetic resonance relaxometry of the normal heart: relationship between collagen content and relaxation times of the four chambers. Magn Reson Imaging.

[14] Burtea C, Gatina R, Stoian G, Mardare M, Dumitru IF, Dragomir CT (1998). Spin-spin relaxation times in myocardial hypertrophy induced by endocrine agents in rat. MAGMA.

[15] Bottomley PA, Foster TH, Argersinger RE, Pfeifer LM (1984). A review of normal tissue hydrogen NMR relaxation times and relaxation mechanisms from 1–100 MHz: dependence on tissue type, NMR frequency, temperature, species, excision, and age. Med Phys.

[16] van Heeswijk RB, Feliciano H, Bongard C, Bonanno G, Coppo S, Lauriers N (2012). Free-breathing 3 T magnetic resonance T2-mapping of the heart. JACC Cardiovasc Imaging.

[17] Baeßler B, Schaarschmidt F, Stehning C, Schnackenburg B, Maintz D, Bunck AC (2015). A systematic evaluation of three different cardiac T2-mapping sequences at 1.5 and 3T in healthy volunteers. Eur J Radiol.

[18] Granitz M, Motloch LJ, Granitz C, Meissnitzer M, Hitzl W, Hergan K (2019). Comparison of native myocardial T1 and T2 mapping at 1.5T and 3T in healthy volunteers : reference values and clinical implications. Wien Klin Wochenschr.

[19] von Knobelsdorff-Brenkenhoff F, Prothmann M, Dieringer MA, Wassmuth R, Greiser A, Schwenke C (2013). Myocardial T1 and T2 mapping at 3 T: reference values, influencing factors and implications. J Cardiovasc Magn Reson.

[20] Hanson CA, Kamath A, Gottbrecht M, Ibrahim S, Salerno M (2020). T2 relaxation times at cardiac MRI in healthy adults: a systematic review and meta-analysis. Radiology.

[21] Varghese J, Craft J, Crabtree CD, Liu Y, Jin N, Chow K (2020). Assessment of cardiac function, blood flow and myocardial tissue relaxation parameters at 035 T. NMR Biomed.

[22] Campbell-Washburn AE, Ramasawmy R, Restivo MC, Bhattacharya I, Basar B, Herzka DA (2019). Opportunities in interventional and diagnostic imaging by using high-performance low-field-strength MRI. Radiology.

[23] Jenista ER, Rehwald WG, Chen E-L, Kim HW, Klem I, Parker MA (2013). Motion and flow insensitive adiabatic T2-preparation module for cardiac MR imaging at 3 Tesla. Magn Reson Med.

[24] Bano W, Feliciano H, Coristine AJ, Stuber M, van Heeswijk RB (2017). On the accuracy and precision of cardiac magnetic resonance T2 mapping: a high-resolution radial study using adiabatic T2 preparation at 3 T. Magn Reson Med.

[25] Huang T-Y, Liu Y-J, Stemmer A, Poncelet BP (2007). T2 measurement of the human myocardium using a T2-prepared transient-state TrueFISP sequence. Magn Reson Med.

[26] Brittain JH, Hu BS, Wright GA, Meyer CH, Macovski A, Nishimura DG (1995). Coronary angiography with magnetization-prepared T2 contrast. Magn Reson Med.

[27] Levitt MH, Freeman R, Frenkiel T (1982). Broadband heteronuclear decoupling. J Magn Reson 1969..

[28] Nezafat R, Stuber M, Ouwerkerk R, Gharib AM, Desai MY, Pettigrew RI (2006). B1-insensitive T2 preparation for improved coronary magnetic resonance angiography at 3 T. Magn Reson Med.

[29] Silver MS, Joseph RI, Hoult DI (1984). Highly selective π2 and π pulse generation. J Magn Reson 1969..

[30] Basha TA, Bellm S, Roujol S, Kato S, Nezafat R (2016). Free-breathing slice-interleaved myocardial T2 mapping with slice-selective T2 magnetization preparation. Magn Reson Med.

[31] van Heeswijk RB, Piccini D, Feliciano H, Hullin R, Schwitter J, Stuber M (2015). Self-navigated isotropic three-dimensional cardiac T2 mapping. Magn Reson Med.

[32] Kim D, Jensen JH, Wu EX, Sheth SS, Brittenham GM (2009). Breathhold multiecho fast spin-echo pulse sequence for accurate R2 measurement in the heart and liver. Magn Reson Med.

[33] Sprinkart AM, Luetkens JA, Träber F, Doerner J, Gieseke J, Schnackenburg B (2015). Gradient Spin Echo (GraSE) imaging for fast myocardial T2 mapping. J Cardiovasc Magn Reson.

[34] Baeßler B, Schaarschmidt F, Stehning C, Schnackenburg B, Maintz D, Bunck AC (2015). Cardiac T2-mapping using a fast gradient echo spin echo sequence—first in vitro and in vivo experience. J Cardiovasc Magn Reson.

[35] Ding H, Fernandez-de-Manuel L, Schär M, Schuleri KH, Halperin H, He L (2015). Three-dimensional whole-heart T2 mapping at 3T. Magn Reson Med.

[36] Akçakaya M, Weingärtner S, Basha TA, Roujol S, Bellm S, Nezafat R (2016). Joint myocardial T1 and T2 mapping using a combination of saturation recovery and T2 -preparation. Magn Reson Med.

[37] Yang H-J, Sharif B, Pang J, Kali A, Bi X, Cokic I (2016). Free-breathing, motion-corrected, highly efficient whole heart T2 mapping at 3T with hybrid radial-cartesian trajectory. Magn Reson Med.

[38] Giri S, Shah S, Xue H, Chung Y-C, Pennell ML, Guehring J (2012). Myocardial T2 mapping with respiratory navigator and automatic nonrigid motion correction. Magn Reson Med.

[39] Henningsson M, Koken P, Stehning C, Razavi R, Prieto C, Botnar RM (2012). Whole-heart coronary MR angiography with 2D self-navigated image reconstruction. Magn Reson Med.

[40] Bustin A, Milotta G, Ismail TF, Neji R, Botnar RM, Prieto C (2020). Accelerated free-breathing whole-heart 3D T2 mapping with high isotropic resolution. Magn Reson Med.

[41] Darçot E, Yerly J, Colotti R, Masci PG, Chaptinel J, Feliciano H (2019). Accelerated and high-resolution cardiac T2 mapping through peripheral k-space sharing. Magn Reson Med.

[42] Zhu D, Ding H, Zviman MM, Halperin H, Schär M, Herzka DA (2021). Accelerating whole-heart 3D T2 mapping: Impact of undersampling strategies and reconstruction techniques. PLoS ONE.

[43] Milotta G, Bustin A, Jaubert O, Neji R, Prieto C, Botnar RM (2020). 3D whole-heart isotropic-resolution motion-compensated joint T1 /T2 mapping and water/fat imaging. Magn Reson Med.

[44] Blume U, Lockie T, Stehning C, Sinclair S, Uribe S, Razavi R (2009). Interleaved T(1) and T(2) relaxation time mapping for cardiac applications. J Magn Reson Imaging JMRI.

[45] Kvernby S, Warntjes MJB, Haraldsson H, Carlhäll C-J, Engvall J, Ebbers T (2014). Simultaneous three-dimensional myocardial T1 and T2 mapping in one breath hold with 3D-QALAS. J Cardiovasc Magn Reson.

[46] Hamilton JI, Jiang Y, Chen Y, Ma D, Lo W-C, Griswold M (2017). MR fingerprinting for rapid quantification of myocardial T1, T2, and proton spin density. Magn Reson Med.

[47] Jaubert O, Cruz G, Bustin A, Hajhosseiny R, Nazir S, Schneider T (2021). T1, T2, and fat fraction cardiac MR fingerprinting: preliminary clinical evaluation. J Magn Reson Imaging JMRI.

[48] Velasco C, Cruz G, Lavin B, Hua A, Fotaki A, Botnar RM (2022). Simultaneous T1, T2, and T1ρ cardiac magnetic resonance fingerprinting for contrast agent-free myocardial tissue characterization. Magn Reson Med.

[49] Snel GJH, van den Boomen M, Hernandez LM, Nguyen CT, Sosnovik DE, Velthuis BK (2020). Cardiovascular magnetic resonance native T2 and T2* quantitative values for cardiomyopathies and heart transplantations: a systematic review and meta-analysis. J Cardiovasc Magn Reson.

[50] Messroghli DR, Moon JC, Ferreira VM, Grosse-Wortmann L, He T, Kellman P (2017). Clinical recommendations for cardiovascular magnetic resonance mapping of T1, T2, T2* and extracellular volume: a consensus statement by the Society for Cardiovascular Magnetic Resonance (SCMR) endorsed by the European Association for Cardiovascular Imaging (EACVI). J Cardiovasc Magn Reson.

[51] Hennig J (1988). Multiecho imaging sequences with low refocusing flip angles. J Magn Reson 1969..

[52] Akçakaya M, Basha TA, Weingärtner S, Roujol S, Berg S, Nezafat R (2015). Improved quantitative myocardial T2 mapping: Impact of the fitting model. Magn Reson Med.

[53] Zabala-Travers S, Choi M, Cheng W-C, Badano A (2015). Effect of color visualization and display hardware on the visual assessment of pseudocolor medical images. Med Phys.

[54] Borland D, Taylor MR (2007). Rainbow color map (still) considered harmful. IEEE Comput Graph Appl.

[55] Kramer CM, Barkhausen J, Bucciarelli-Ducci C, Flamm SD, Kim RJ, Nagel E (2020). Standardized cardiovascular magnetic resonance imaging (CMR) protocols: 2020 update. J Cardiovasc Magn Reson.

[56] Tessa C, Diciotti S, Landini N, Lilli A, Del Meglio J, Salvatori L (2015). Myocardial T1 and T2 mapping in diastolic and systolic phase. Int J Cardiovasc Imaging.

[57] Schulz-Menger J, Bluemke DA, Bremerich J, Flamm SD, Fogel MA, Friedrich MG (2020). Standardized image interpretation and post-processing in cardiovascular magnetic resonance—2020 update. J Cardiovasc Magn Reson.

[58] Selvadurai BSN, Puntmann VO, Bluemke DA, Ferrari VA, Friedrich MG, Kramer CM (2018). Definition of left ventricular segments for cardiac magnetic resonance imaging. JACC Cardiovasc Imaging.

[59] Bönner F, Janzarik N, Jacoby C, Spieker M, Schnackenburg B, Range F (2015). Myocardial T2 mapping reveals age- and sex-related differences in volunteers. J Cardiovasc Magn Reson.

[60] Luetkens JA, Voigt M, Faron A, Isaak A, Mesropyan N, Dabir D (2020). Influence of hydration status on cardiovascular magnetic resonance myocardial T1 and T2 relaxation time assessment: an intraindividual study in healthy subjects. J Cardiovasc Magn Reson.

[61] Taylor AJ, Salerno M, Dharmakumar R, Jerosch-Herold M (2016). T1 mapping: basic techniques and clinical applications. JACC Cardiovasc Imaging.

[62] Messroghli DR, Walters K, Plein S, Sparrow P, Friedrich MG, Ridgway JP (2007). Myocardial T1 mapping: application to patients with acute and chronic myocardial infarction. Magn Reson Med.

[63] Ugander M, Oki AJ, Hsu L-Y, Kellman P, Greiser A, Aletras AH (2012). Extracellular volume imaging by magnetic resonance imaging provides insights into overt and sub-clinical myocardial pathology. Eur Heart J.

[64] Kellman P, Wilson JR, Xue H, Bandettini WP, Shanbhag SM, Druey KM (2012). Extracellular volume fraction mapping in the myocardium, part 2: initial clinical experience. J Cardiovasc Magn Reson.

[65] Ferreira VM, Piechnik SK, Dall’Armellina E, Karamitsos TD, Francis JM, Ntusi N (2013). T(1) mapping for the diagnosis of acute myocarditis using CMR: comparison to T2-weighted and late gadolinium enhanced imaging. JACC Cardiovasc Imaging.

[66] Feng Y, He T, Carpenter J-P, Jabbour A, Alam MH, Gatehouse PD (2013). In vivo comparison of myocardial T1 with T2 and T2* in thalassaemia major. J Magn Reson Imaging JMRI.

[67] Sado DM, Maestrini V, Piechnik SK, Banypersad SM, White SK, Flett AS (2015). Noncontrast myocardial T1 mapping using cardiovascular magnetic resonance for iron overload. J Magn Reson Imaging JMRI.

[68] Wang L, Yuan J, Zhang S-J, Gao M, Wang Y-C, Wang Y-X (2016). Myocardial T1 rho mapping of patients with end-stage renal disease and its comparison with T1 mapping and T2 mapping: a feasibility and reproducibility study. J Magn Reson Imaging JMRI.

[69] Han Y, Liimatainen T, Gorman RC, Witschey WRT (2014). Assessing myocardial disease using T1ρ MRI. Curr Cardiovasc Imaging Rep.

[70] Witschey WRT, Pilla JJ, Ferrari G, Koomalsingh K, Haris M, Hinmon R (2010). Rotating frame spin lattice relaxation in a swine model of chronic, left ventricular myocardial infarction. Magn Reson Med.

[71] Verhaert D, Thavendiranathan P, Giri S, Mihai G, Rajagopalan S, Simonetti OP (2011). Direct T2 quantification of myocardial edema in acute ischemic injury. JACC Cardiovasc Imaging.

[72] Thavendiranathan P, Walls M, Giri S, Verhaert D, Rajagopalan S, Moore S (2012). Improved detection of myocardial involvement in acute inflammatory cardiomyopathies using T2 mapping. Circ Cardiovasc Imaging.

[73] Kali A, Tang RLQ, Kumar A, Min JK, Dharmakumar R (2013). Detection of acute reperfusion myocardial hemorrhage with cardiac MR imaging: T2 versus T2. Radiology.

[74] Friedrich MG, Niendorf T, Schulz-Menger J, Gross CM, Dietz R (2003). Blood oxygen level-dependent magnetic resonance imaging in patients with stress-induced angina. Circulation.

[75] Anderson LJ, Holden S, Davis B, Prescott E, Charrier CC, Bunce NH (2001). Cardiovascular T2-star (T2*) magnetic resonance for the early diagnosis of myocardial iron overload. Eur Heart J.

[76] Carpenter J-P, He T, Kirk P, Roughton M, Anderson LJ, de Noronha SV (2011). On T2* magnetic resonance and cardiac iron. Circulation.

[77] Bordonaro V, Bivort D, Dresselaers T, De Langhe E, Bogaert J, Symons R (2020). Myocardial T1 mapping and extracellular volume quantification as novel biomarkers in risk stratification of patients with systemic sclerosis. Clin Radiol.

[78] Gastl M, Lachmann V, Christidi A, Janzarik N, Veulemans V, Haberkorn S (2020). Cardiac magnetic resonance T2 mapping and feature tracking in athlete’s heart and HCM. Eur Radiol.

[79] Pavon AG, Georgiopoulos G, Vincenti G, Muller O, Monney P, Berchier G (2020). Head-to-head comparison of multiple cardiovascular magnetic resonance techniques for the detection and quantification of intramyocardial haemorrhage in patients with ST-elevation myocardial infarction. Eur Radiol.

[80] Flamée L, Symons R, Degtiarova G, Dresselaers T, Gheysens O, Wuyts W (2020). Prognostic value of cardiovascular magnetic resonance in patients with biopsy-proven systemic sarcoidosis. Eur Radiol.

[81] Altaha MA, Nolan M, Marwick TH, Somerset E, Houbois C, Amir E (2020). Can quantitative CMR tissue characterization adequately identify cardiotoxicity during chemotherapy?: Impact of temporal and observer variability. JACC Cardiovasc Imaging.

[82] Dolan RS, Rahsepar AA, Blaisdell J, Suwa K, Ghafourian K, Wilcox JE (2019). Multiparametric cardiac magnetic resonance imaging can detect acute cardiac allograft rejection after heart transplantation. JACC Cardiovasc Imaging.

[83] Blaszczyk E, Grieben U, von Knobelsdorff-Brenkenhoff F, Kellman P, Schmacht L, Funk S (2019). Subclinical myocardial injury in patients with Facioscapulohumeral muscular dystrophy 1 and preserved ejection fraction—assessment by cardiovascular magnetic resonance. J Cardiovasc Magn Reson.

[84] Kotecha T, Martinez-Naharro A, Treibel TA, Francis R, Nordin S, Abdel-Gadir A (2018). Myocardial edema and prognosis in amyloidosis. J Am Coll Cardiol.

[85] Fehrmann A, Treutlein M, Rudolph T, Rudolph V, Weiss K, Giese D (2018). Myocardial T1 and T2 mapping in severe aortic stenosis: potential novel insights into the pathophysiology of myocardial remodelling. Eur J Radiol.

[86] Luetkens JA, Schlesinger-Irsch U, Kuetting DL, Dabir D, Homsi R, Doerner J (2017). Feature-tracking myocardial strain analysis in acute myocarditis: diagnostic value and association with myocardial oedema. Eur Radiol.

[87] Krittayaphong R, Zhang S, Saiviroonporn P, Viprakasit V, Tanapibunpon P, Komoltri C (2017). Detection of cardiac iron overload with native magnetic resonance T1 and T2 mapping in patients with thalassemia. Int J Cardiol.

[88] Mayr A, Kitterer D, Latus J, Steubing H, Henes J, Vecchio F (2016). Evaluation of myocardial involvement in patients with connective tissue disorders: a multi-parametric cardiovascular magnetic resonance study. J Cardiovasc Magn Reson.

[89] Diederichsen LP, Simonsen JA, Diederichsen ACP, Kim WY, Hvidsten S, Hougaard M (2015). Cardiac abnormalities assessed by non-invasive techniques in patients with newly diagnosed idiopathic inflammatory myopathies. Clin Exp Rheumatol.

[90] Spieker M, Katsianos E, Gastl M, Behm P, Horn P, Jacoby C (2018). T2 mapping cardiovascular magnetic resonance identifies the presence of myocardial inflammation in patients with dilated cardiomyopathy as compared to endomyocardial biopsy. Eur Heart J Cardiovasc Imaging.

[91] Willerson JT, Scales F, Mukherjee A, Platt M, Templeton GH, Fink GS (1977). Abnormal myocardial fluid retention as an early manifestation of ischemic injury. Am J Pathol.

[92] Wisenberg G, Prato FS, Carroll SE, Turner KL, Marshall T (1988). Serial nuclear magnetic resonance imaging of acute myocardial infarction with and without reperfusion. Am Heart J.

[93] Tahir E, Sinn M, Bohnen S, Avanesov M, Säring D, Stehning C (2017). Acute versus chronic myocardial infarction: diagnostic accuracy of quantitative native T1 and T2 mapping versus assessment of edema on standard T2-weighted cardiovascular MR images for differentiation. Radiology.

[94] Layland J, Rauhalammi S, Lee MMY, Ahmed N, Carberry J, Teng Yue May V (2017). Diagnostic accuracy of 30-T magnetic resonance T1 and T2 mapping and T2-weighted dark-blood imaging for the infarct-related coronary artery in non–ST-segment elevation myocardial infarction. J Am Heart Assoc Cardiovasc Cerebrovasc Dis..

[95] Wang G, Yang H-J, Kali A, Cokic I, Tang R, Xie G (2019). Influence of myocardial hemorrhage on staging of reperfused myocardial infarctions with T2 cardiac magnetic resonance imaging: insights into the dependence on infarction type with ex vivo validation. JACC Cardiovasc Imaging.

[96] Fernández-Jiménez R, Sánchez-González J, Agüero J, García-Prieto J, López-Martín GJ, García-Ruiz JM (2015). Myocardial edema after ischemia/reperfusion is not stable and follows a bimodal pattern: imaging and histological tissue characterization. J Am Coll Cardiol.

[97] Kim HW, Van Assche L, Jennings RB, Wince WB, Jensen CJ, Rehwald WG (2015). Relationship of T2-weighted MRI myocardial hyperintensity and the ischemic area-at-risk. Circ Res.

[98] Bulluck H, White SK, Rosmini S, Bhuva A, Treibel TA, Fontana M (2015). T1 mapping and T2 mapping at 3T for quantifying the area-at-risk in reperfused STEMI patients. J Cardiovasc Magn Reson.

[99] Langhans B, Nadjiri J, Jähnichen C, Kastrati A, Martinoff S, Hadamitzky M (2014). Reproducibility of area at risk assessment in acute myocardial infarction by T1- and T2-mapping sequences in cardiac magnetic resonance imaging in comparison to Tc99m-sestamibi SPECT. Int J Cardiovasc Imaging.

[100] Fernández-Jiménez R, Barreiro-Pérez M, Martin-García A, Sánchez-González J, Agüero J, Galán-Arriola C (2017). Dynamic edematous response of the human heart to myocardial infarction: implications for assessing myocardial area at risk and salvage. Circulation.

[101] Masci P-G, Pavon AG, Muller O, Iglesias J-F, Vincenti G, Monney P (2018). Relationship between CMR-derived parameters of ischemia/reperfusion injury and the timing of CMR after reperfused ST-segment elevation myocardial infarction. J Cardiovasc Magn Reson.

[102] Carrick D, Haig C, Ahmed N, McEntegart M, Petrie MC, Eteiba H (2016). Myocardial hemorrhage after acute reperfused ST-segment-elevation myocardial infarction: relation to microvascular obstruction and prognostic significance. Circ Cardiovasc Imaging.

[103] Nakamori S, Fahmy A, Jang J, El-Rewaidy H, Neisius U, Berg S (2020). Changes in myocardial native T1 and T2 after exercise stress: a noncontrast CMR pilot study. JACC Cardiovasc Imaging.

[104] Nickander J, Themudo R, Thalén S, Sigfridsson A, Xue H, Kellman P (2019). The relative contributions of myocardial perfusion, blood volume and extracellular volume to native T1 and native T2 at rest and during adenosine stress in normal physiology. J Cardiovasc Magn Reson.

[105] Von Knobelsdorff-Brenkenhoff F, Schüler J, Dogangüzel S, Dieringer MA, Rudolph A, Greiser A (2017). Detection and monitoring of acute myocarditis applying quantitative cardiovascular magnetic resonance. Circ Cardiovasc Imaging.

[106] Grn S, Schumm J, Greulich S, Wagner A, Schneider S, Bruder O (2012). Long-term follow-up of biopsy-proven viral myocarditis: predictors of mortality and incomplete recovery. J Am Coll Cardiol.

[107] Spieker M, Haberkorn S, Gastl M, Behm P, Katsianos S, Horn P (2017). Abnormal T2 mapping cardiovascular magnetic resonance correlates with adverse clinical outcome in patients with suspected acute myocarditis. J Cardiovasc Magn Reson.

[108] Lurz P, Luecke C, Eitel I, Föhrenbach F, Frank C, Grothoff M (2016). Comprehensive cardiac magnetic resonance imaging in patients with suspected myocarditis: the MyoRacer-Trial. J Am Coll Cardiol.

[109] Tschöpe C, Ammirati E, Bozkurt B, Caforio ALP, Cooper LT, Felix SB (2020). Myocarditis and inflammatory cardiomyopathy: current evidence and future directions. Nat Rev Cardiol.

[110] Bönner F, Spieker M, Haberkorn S, Jacoby C, Flögel U, Schnackenburg B (2016). Myocardial T2 mapping increases noninvasive diagnostic accuracy for biopsy-proven myocarditis. JACC Cardiovasc Imaging.

[111] P Caforio AL, Pankuweit S, Arbustini E, Basso C, Gimeno-Blanes J, Felix SB, et al. Current state of knowledge on aetiology, diagnosis, management, and therapy of myocarditis: a position statement of the European Society of Cardiology Working Group on Myocardial and Pericardial Diseases. http://eurheartj.oxfordjournals.org/. 10.1093/eurheartj/eht210 23824828

[112] Ferreira VM, Schulz-Menger J, Holmvang G, Kramer CM, Carbone I, Sechtem U (2018). Cardiovascular magnetic resonance in nonischemic myocardial inflammation: expert recommendations. J Am Coll Cardiol.

[113] Puntmann VO, Isted A, Hinojar R, Foote L, Carr-White G, Nagel E (2017). T1 and T2 mapping in recognition of early cardiac involvement in systemic sarcoidosis. Radiology.

[114] Bohnen S, Radunski UK, Lund GK, Kandolf R, Stehning C, Schnackenburg B (2015). Performance of t1 and t2 mapping cardiovascular magnetic resonance to detect active myocarditis in patients with recent-onset heart failure. Circ Cardiovasc Imaging.

[115] Han Y, Chen T, Bryant J, Bucciarelli-Ducci C, Dyke C, Elliott MD (2020). Society for Cardiovascular Magnetic Resonance (SCMR) guidance for the practice of cardiovascular magnetic resonance during the COVID-19 pandemic. J Cardiovasc Magn Reson.

[116] Allen BD, Wong TC, Bucciarelli-Ducci C, Bryant J, Chen T, Dall’Armellina E (2020). Society for Cardiovascular Magnetic Resonance (SCMR) guidance for re-activation of cardiovascular magnetic resonance practice after peak phase of the COVID-19 pandemic. J Cardiovasc Magn Reson.

[117] Kelle S, Bucciarelli-Ducci C, Judd RM, Kwong RY, Simonetti O, Plein S (2020). Society for Cardiovascular Magnetic Resonance (SCMR) recommended CMR protocols for scanning patients with active or convalescent phase COVID-19 infection. J Cardiovasc Magn Reson.

[118] Puntmann VO, Carerj ML, Wieters I, Fahim M, Arendt C, Hoffmann J (2020). Outcomes of cardiovascular magnetic resonance imaging in patients recently recovered from coronavirus disease 2019 COVID-19. JAMA Cardiol..

[119] Joy G, Artico J, Kurdi H, Seraphim A, Lau C, Thornton GD (2021). Prospective case-control study of cardiovascular abnormalities 6 months following mild COVID-19 in healthcare workers. JACC Cardiovasc Imaging.

[120] Rajpal S, Tong MS, Borchers J, Zareba KM, Obarski TP, Simonetti OP (2020). Cardiovascular magnetic resonance findings in competitive athletes recovering from COVID-19 infection. JAMA Cardiol..

[121] Clark DE, Parikh A, Dendy JM, Diamond AB, George-Durrett K, Fish FA, Slaughter JC, Fitch W, Hughes SG, Soslow JH. COVID-19 myocardial pathology evaluation in athletes with cardiac magnetic resonance (COMPETE CMR). Circulation. 2021;143:609–612. 10.1161/CIRCULATIONAHA.120.052573. 10.1161/CIRCULATIONAHA.120.052573 PMC7864610 33332151

[122] Vago H, Szabo L, Dohy Z, Merkely B. Cardiac magnetic resonance findings in patients recovered from COVID-19. JACC Cardiovasc Imaging. 2020;S1936878X20310214. 10.1016/j.jcmg.2020.11.014 PMC7837171 33341416

[123] Brito D, Meester S, Yanamala N, Patel HB, Balcik BJ, Casaclang-Verzosa G (2020). High prevalence of pericardial involvement in college student athletes recovering from COVID-19. JACC Cardiovasc Imaging.

[124] Phelan D, Kim JH, Elliott MD, Wasfy MM, Cremer P, Johri AM (2020). Screening of potential cardiac involvement in competitive athletes recovering from COVID-19. Jacc Cardiovasc Imaging.

[125] Gonzalez JA, Kramer CM (2015). Role of imaging techniques for diagnosis, prognosis and management of heart failure patients: cardiac magnetic resonance. Curr Heart Fail Rep.

[126] Kindermann I, Barth C, Mahfoud F, Ukena C, Lenski M, Yilmaz A (2012). Update on myocarditis. J Am Coll Cardiol.

[127] Barison A, Aimo A, Todiere G, Grigoratos C, Aquaro GD, Emdin M (2020). Cardiovascular magnetic resonance for the diagnosis and management of heart failure with preserved ejection fraction. Heart Fail Rev.

[128] Leong DP, De Pasquale CG, Selvanayagam JB (2010). Heart failure with normal ejection fraction: the complementary roles of echocardiography and CMR imaging. JACC Cardiovasc Imaging.

[129] Doeblin P, Hashemi D, Tanacli R, Lapinskas T, Gebker R, Stehning C (2019). CMR tissue characterization in patients with HFmrEF. J Clin Med.

[130] Verbrugge FH, Bertrand PB, Willems E, Gielen E, Mullens W, Giri S (2017). Global myocardial oedema in advanced decompensated heart failure. Eur Heart J Cardiovasc Imaging.

[131] Emrich T, Hahn F, Fleischmann D, Halfmann MC, Düber C, Varga-Szemes A (2020). T1 and T2 mapping to detect chronic inflammation in cardiac magnetic resonance imaging in heart failure with reduced ejection fraction. ESC Heart Fail.

[132] Nishii T, Kono AK, Shigeru M, Takamine S, Fujiwara S, Kyotani K (2014). Cardiovascular magnetic resonance T2 mapping can detect myocardial edema in idiopathic dilated cardiomyopathy. Int J Cardiovasc Imaging.

[133] Mordi I, Carrick D, Bezerra H, Tzemos N (2016). T1 and T2 mapping for early diagnosis of dilated non-ischaemic cardiomyopathy in middle-aged patients and differentiation from normal physiological adaptation. Eur Heart J Cardiovasc Imaging.

[134] Xu Y, Li W, Wan K, Liang Y, Jiang X, Wang J (2020). Myocardial tissue reverse remodeling after guideline-directed medical therapy in idiopathic dilated cardiomyopathy. Circ Heart Fail.

[135] Huang L, Ran L, Zhao P, Tang D, Han R, Ai T (2019). MRI native T1 and T2 mapping of myocardial segments in hypertrophic cardiomyopathy: tissue remodeling mani. Br J Radiol.

[136] Arcari L, Hinojar R, Engel J, Freiwald T, Platschek S, Zainal H (2020). Native T1 and T2 provide distinctive signatures in hypertrophic cardiac conditions—comparison of uremic, hypertensive and hypertrophic cardiomyopathy. Int J Cardiol.

[137] Baig M, Galazka P, Dakwar O, Syed SA, Sawlani R, Shahir K (2021). Prevalence of myocardial edema with t2 mapping in hypertrophic cardiomyopathy. J Am Coll Cardiol.

[138] Khush KK, Cherikh WS, Chambers DC, Harhay MO, Hayes D, Hsich E (2019). The international thoracic organ transplant registry of the international society for heart and lung transplantation: thirty-sixth adult heart transplantation report—2019; focus theme: donor and recipient size match. J Heart Lung Transplant.

[139] Lund LH, Edwards LB, Dipchand AI, Goldfarb S, Kucheryavaya AY, Levvey BJ (2016). The Registry of the International Society for Heart and Lung Transplantation: thirty-third adult heart transplantation report-2016; focus theme: primary diagnostic indications for transplant. J Heart Lung Transplant.

[140] Hamour IM, Burke MM, Bell AD, Panicker MG, Banerjee R, Banner NR (2008). Limited utility of endomyocardial biopsy in the first year after heart transplantation. Transplantation.

[141] Aherne T, Tscholakoff D, Finkbeiner W, Sechtem U, Derugin N, Yee E (1986). Magnetic resonance imaging of cardiac transplants: the evaluation of rejection of cardiac allografts with and without immunosuppression. Circulation.

[142] Butler CR, Thompson R, Haykowsky M, Toma M, Paterson I (2009). Cardiovascular magnetic resonance in the diagnosis of acute heart transplant rejection: a review. J Cardiovasc Magn Reson.

[143] Marie PY, Angioï M, Carteaux JP, Escanye JM, Mattei S, Tzvetanov K (2001). Detection and prediction of acute heart transplant rejection with the myocardial T2 determination provided by a black-blood magnetic resonance imaging sequence. J Am Coll Cardiol.

[144] Marie PY, Carteaux JP, Angioï M, Marwan NS, Tzvetanov K, Escanye JM (1998). Detection and prediction of acute heart transplant rejection: preliminary results on the clinical use of a “black blood” magnetic resonance imaging sequence. Transplant Proc.

[145] Wisenberg G, Pflugfelder PW, Kostuk WJ, McKenzie FN, Prato FS (1987). Diagnostic applicability of magnetic resonance imaging in assessing human cardiac allograft rejection. Am J Cardiol.

[146] Usman AA, Taimen K, Wasielewski M, McDonald J, Shah S, Giri S (2012). Cardiac magnetic resonance T2 mapping in the monitoring and follow-up of acute cardiac transplant rejection: a pilot study. Circ Cardiovasc Imaging.

[147] Miller CA, Naish JH, Shaw SM, Yonan N, Williams SG, Clark D (2014). Multiparametric cardiovascular magnetic resonance surveillance of acute cardiac allograft rejection and characterisation of transplantation-associated myocardial injury: a pilot study. J Cardiovasc Magn Reson.

[148] Vermes E, Pantaléon C, Auvet A, Cazeneuve N, Machet MC, Delhommais A (2018). Cardiovascular magnetic resonance in heart transplant patients: diagnostic value of quantitative tissue markers: T2 mapping and extracellular volume fraction, for acute rejection diagnosis. J Cardiovasc Magn Reson.

[149] Ridouani F, Damy T, Tacher V, Derbel H, Legou F, Sifaoui I (2018). Myocardial native T2 measurement to differentiate light-chain and transthyretin cardiac amyloidosis and assess prognosis. J Cardiovasc Magn Reson.

[150] Banypersad SM (2019). The evolving role of cardiovascular magnetic resonance imaging in the evaluation of systemic amyloidosis. Magn Reson Insights..

[151] Slivnick JA, Tong MS, Nagaraja HN, Elamin MB, Wallner A, O’Brien A (2020). Novel cardiovascular magnetic resonance model to distinguish immunoglobulin light chain from transthyretin cardiac amyloidosis. JACC Cardiovasc Imaging.

[152] Velangi PS, Chen KHA, Kazmirczak F, Okasha O, von Wald L, Roukoz H (2020). Right ventricular abnormalities on cardiovascular magnetic resonance imaging in patients with sarcoidosis. JACC Cardiovasc Imaging.

[153] Patel AR, Patel H (2020). Cardiac sarcoidosis: remembering the forgotten right ventricle—PubMed. JACC Cardiovasc Imaging.

[154] Bravo PE, Singh A, Di CMF, Blankstein R (2019). Advanced cardiovascular imaging for the evaluation of cardiac sarcoidosis HHS Public Access. J Nucl Cardiol.

[155] Hulten E, Aslam S, Osborne M, Abbasi S, Bittencourt MS, Blankstein R (2016). Cardiac sarcoidosis-state of the art review. Cardiovasc Diagn Ther.

[156] Crouser ED, Ono C, Tran T, He X, Raman SV (2014). Improved detection of cardiac sarcoidosis using magnetic resonance with myocardial T2 mapping. Am J Respir Crit Care Med.

[157] Simon G, Daniel K, Joerg L, Eissa A, Hannah S, Philipp K (2016). Comprehensive cardiovascular magnetic resonance assessment in patients with sarcoidosis and preserved left ventricular ejection fraction. Circ Cardiovasc Imaging.

[158] Crouser ED, Ruden E, Julian MW, Raman SV (2016). Resolution of abnormal cardiac MRI T2 signal following immune suppression for cardiac sarcoidosis. J Investig Med.

[159] Aronow WS (2018). Management of cardiac hemochromatosis. Arch Med Sci.

[160] Čelutkienė J, Plymen CM, Flachskampf FA, de Boer RA, Grapsa J, Manka R (2018). Innovative imaging methods in heart failure: a shifting paradigm in cardiac assessment. Position statement on behalf of the Heart Failure Association of the European Society of Cardiology. Eur J Heart Fail.

[161] Guo H, Au WY, Cheung JS, Kim D, Jensen JH, Khong PL (2009). Myocardial T2 quantitation in patients with iron overload at 3 Tesla. J Magn Reson Imaging.

[162] Camargo GC, Rothstein T, Junqueira FP, Fernandes E, Greiser A, Strecker R (2016). Comparison of myocardial T1 and T2 values in 3 T with T2* in 1.5 T in patients with iron overload and controls. Int J Hematol.

[163] Krittayaphong R, Zhang S, Saiviroonporn P, Viprakasit V, Tanapibunpon P, Rerkudom B (2019). Assessment of cardiac iron overload in thalassemia with MRI on 3.0-T: high-field T1, T2, and T2* quantitative parametric mapping in comparison to T2* on 1.5-T. JACC Cardiovasc Imaging.

[164] Kritsaneepaiboon S, Ina N, Chotsampancharoen T, Roymanee S, Cheewatanakornkul S (2018). The relationship between myocardial and hepatic T2 and T2* at 1.5T and 3T MRI in normal and iron-overloaded patients. Acta Radiol.

[165] Militaru S, Ginghina C, Popescu BA, Saftoiu A, Linhart A, Jurcut R (2018). Multimodality imaging in Fabry cardiomyopathy: from early diagnosis to therapeutic targets. Eur Heart J Cardiovasc Imaging.

[166] Knott KD, Augusto JB, Nordin S, Kozor R, Camaioni C, Xue H (2019). Quantitative myocardial perfusion in Fabry disease. Circ Cardiovasc Imaging.

[167] Messalli G, Imbriaco M, Avitabile G, Russo R, Iodice D, Spinelli L (2012). Role of cardiac MRI in evaluating patients with Anderson-Fabry disease: assessing cardiac effects of long-term enzyme replacement therapy. Radiol Med (Torino).

[168] Nordin S, Kozor R, Bulluck H, Castelletti S, Rosmini S, Abdel-Gadir A (2016). Cardiac fabry disease with late gadolinium enhancement is a chronic inflammatory cardiomyopathy. J Am Coll Cardiol.

[169] Kribben A, Witzke O, Hillen U, Barkhausen J, Daul AE, Erbel R (2009). Nephrogenic systemic fibrosis: pathogenesis, diagnosis, and therapy. J Am Coll Cardiol.

[170] Woolen SA, Shankar PR, Gagnier JJ, MacEachern MP, Singer L, Davenport MS (2020). Risk of nephrogenic systemic fibrosis in patients with stage 4 or 5 chronic kidney disease receiving a group II gadolinium-based contrast agent. JAMA Intern Med.

[171] Zhang Y, Corona-Villalobos CP, Kiani AN, Eng J, Kamel IR, Zimmerman SL (2015). Myocardial T2 mapping by cardiovascular magnetic resonance reveals subclinical myocardial inflammation in patients with systemic lupus erythematosus. Int J Cardiovasc Imaging.

[172] Mavrogeni S, Bratis K, Markussis V, Spargias C, Papadopoulou E, Papamentzelopoulos S (2013). The diagnostic role of cardiac magnetic resonance imaging in detecting myocardial inflammation in systemic lupus erythematosus. Differentiation from viral myocarditis. Lupus.

[173] Hinojar R, Foote L, Sangle S, Marber M, Mayr M, Carr-White G (2016). Native T1 and T2 mapping by CMR in lupus myocarditis: disease recognition and response to treatment. Int J Cardiol.

[174] Winau L, Hinojar Baydes R, Braner A, Drott U, Burkhardt H, Sangle S (2018). High-sensitive troponin is associated with subclinical imaging biosignature of inflammatory cardiovascular involvement in systemic lupus erythematosus. Ann Rheum Dis.

[175] Mavrogeni SI, Sfikakis PP, Markousis-Mavrogenis G, Bournia V-K, Poulos G, Koutsogeorgopoulou L (2019). Cardiovascular magnetic resonance imaging pattern in patients with autoimmune rheumatic diseases and ventricular tachycardia with preserved ejection fraction. Int J Cardiol.

[176] Diao K-Y, Yang Z-G, Xu H-Y, Liu X, Zhang Q, Shi K (2016). Histologic validation of myocardial fibrosis measured by T1 mapping: a systematic review and meta-analysis. J Cardiovasc Magn Reson.

[177] Poindron V, Chatelus E, Canuet M, Gottenberg J-E, Arnaud L, Gangi A (2020). T1 mapping cardiac magnetic resonance imaging frequently detects subclinical diffuse myocardial fibrosis in systemic sclerosis patients. Semin Arthritis Rheum.

[178] Markousis-Mavrogenis G, Bournia V-K, Panopoulos S, Koutsogeorgopoulou L, Kanoupakis G, Apostolou D (2019). Cardiovascular magnetic resonance identifies high-risk systemic sclerosis patients with normal echocardiograms and provides incremental prognostic value. Diagnostics Basel Switz..

[179] Huber AT, Lamy J, Bravetti M, Bouazizi K, Bacoyannis T, Roux C (2019). Comparison of MR T1 and T2 mapping parameters to characterize myocardial and skeletal muscle involvement in systemic idiopathic inflammatory myopathy (IIM). Eur Radiol.

[180] Huber AT, Bravetti M, Lamy J, Bacoyannis T, Roux C, de Cesare A (2018). Non-invasive differentiation of idiopathic inflammatory myopathy with cardiac involvement from acute viral myocarditis using cardiovascular magnetic resonance imaging T1 and T2 mapping. J Cardiovasc Magn Reson.

[181] Xu Y, Sun J, Wan K, Yu L, Wang J, Li W (2020). Multiparametric cardiovascular magnetic resonance characteristics and dynamic changes in myocardial and skeletal muscles in idiopathic inflammatory cardiomyopathy. J Cardiovasc Magn Reson.

[182] Greulich S, Mayr A, Kitterer D, Latus J, Henes J, Vecchio F (2017). Advanced myocardial tissue characterisation by a multi-component CMR protocol in patients with rheumatoid arthritis. Eur Radiol.

[183] Greulich S, Mayr A, Kitterer D, Latus J, Henes J, Steubing H (2017). T1 and T2 mapping for evaluation of myocardial involvement in patients with ANCA-associated vasculitides. J Cardiovasc Magn Reson.

[184] Dweck MR, Boon NA, Newby DE (2012). Calcific aortic stenosis: a disease of the valve and the myocardium. J Am Coll Cardiol.

[185] Bing R, Cavalcante JL, Everett RJ, Clavel M-A, Newby DE, Dweck MR (2019). Imaging and impact of myocardial fibrosis in aortic stenosis. Jacc Cardiovasc Imaging.

[186] Park S-J, Cho SW, Kim SM, Ahn J, Carriere K, Jeong DS (2019). Assessment of myocardial fibrosis using multimodality imaging in severe aortic stenosis: comparison with histologic fibrosis. JACC Cardiovasc Imaging.

[187] Lurz JA, Luecke C, Lang D, Besler C, Rommel K-P, Klingel K (2018). CMR-derived extracellular volume fraction as a marker for myocardial fibrosis: the importance of coexisting myocardial inflammation. JACC Cardiovasc Imaging.

[188] Gastl M, Behm P, Haberkorn S, Holzbach L, Veulemans V, Jacoby C (2018). Role of T2 mapping in left ventricular reverse remodeling after TAVR. Int J Cardiol.

[189] Wang J, Zhao H, Wang Y, Herrmann HC, Witschey WRT, Han Y (2018). Native T1 and T2 mapping by cardiovascular magnetic resonance imaging in pressure overloaded left and right heart diseases. J Thorac Dis.

[190] Feingold B, Mahle WT, Auerbach S, Clemens P, Domenighetti AA, Jefferies JL (2017). Management of cardiac involvement associated with neuromuscular diseases: a scientific statement from the American Heart Association. Circulation.

[191] Nishimura T, Yanagisawa A, Sakata H, Sakata K, Shimoyama K, Ishihara T (2001). Thallium-201 single photon emission computed tomography (SPECT) in patients with duchenne’s progressive muscular dystrophy: a histopathologic correlation study. Jpn Circ J.

[192] Mavrogeni S, Papavasiliou A, Giannakopoulou K, Markousis-Mavrogenis G, Pons MR, Karanasios E (2017). Oedema-fibrosis in Duchenne muscular dystrophy: role of cardiovascular magnetic resonance imaging. Eur J Clin Invest.

[193] Wansapura JP, Hor KN, Mazur W, Fleck R, Hagenbuch S, Benson DW (2010). Left ventricular T2 distribution in Duchenne muscular dystrophy. J Cardiovasc Magn Reson.

[194] Gaur L, Hanna A, Bandettini WP, Fischbeck KH, Arai AE, Mankodi A (2016). Upper arm and cardiac magnetic resonance imaging in Duchenne muscular dystrophy. Ann Clin Transl Neurol.

[195] Schmacht L, Traber J, Grieben U, Utz W, Dieringer MA, Kellman P (2016). Cardiac involvement in myotonic dystrophy type 2 patients with preserved ejection fraction: detection by cardiovascular magnetic resonance. Circ Cardiovasc Imaging.

[196] La Gerche A, Taylor AJ, Prior DL (2009). Athlete’s heart: the potential for multimodality imaging to address the critical remaining questions. JACC Cardiovasc Imaging.

[197] Doerner J, Eichhorn L, Luetkens JA, Lunkenheimer JN, Albers J, Nadal J (2018). Effects of repetitive prolonged breath-hold in elite divers on myocardial fibrosis and cerebral morphology. Eur J Radiol.

[198] Tahir E, Scherz B, Starekova J, Muellerleile K, Fischer R, Schoennagel B (2020). Acute impact of an endurance race on cardiac function and biomarkers of myocardial injury in triathletes with and without myocardial fibrosis. Eur J Prev Cardiol.

[199] Małek ŁA, Barczuk-Falęcka M, Werys K, Czajkowska A, Mróz A, Witek K (2019). Cardiovascular magnetic resonance with parametric mapping in long-term ultra-marathon runners. Eur J Radiol.

[200] Markousis-Mavrogenis G, Giannakopoulou A, Andreou N, Papadopoulos G, Vartela V, Kolovou G (2020). Cardiovascular magnetic resonance clarifies arrhythmogenicity in asymptomatic young athletes with ventricular arrhythmias undergoing pre-participation evaluation. Exp Ther Med.

[201] Abdel-Aty H, Cocker M, Friedrich MG (2009). Myocardial edema is a feature of Tako-Tsubo cardiomyopathy and is related to the severity of systolic dysfunction: insights from T2-weighted cardiovascular magnetic resonance. Int J Cardiol.

[202] Pelliccia F, Kaski JC, Crea F, Camici PG (2017). Pathophysiology of Takotsubo Syndrome. Circulation.

[203] Eitel I, Lücke C, Grothoff M, Sareban M, Schuler G, Thiele H (2010). Inflammation in takotsubo cardiomyopathy: insights from cardiovascular magnetic resonance imaging. Eur Radiol.

[204] Aikawa Y, Noguchi T, Morita Y, Tateishi E, Kono A, Miura H (2019). Clinical impact of native T1 mapping for detecting myocardial impairment in takotsubo cardiomyopathy. Eur Heart J Cardiovasc Imaging.

[205] Dabir D, Luetkens J, Kuetting DLR, Feisst A, Isaak A, Schild HH (2019). Cardiac magnetic resonance including parametric mapping in acute Takotsubo syndrome: preliminary findings. Eur J Radiol.

[206] Vermes E, Berradja N, Saab I, Genet T, Bertrand P, Pucheux J (2020). Cardiac magnetic resonance for assessment of cardiac involvement in Takotsubo syndrome: do we still need contrast administration?. Int J Cardiol.

[207] Hilfiker-Kleiner D, Haghikia A, Nonhoff J, Bauersachs J (2015). Peripartum cardiomyopathy: current management and future perspectives. Eur Heart J.

[208] Schelbert EB, Elkayam U, Cooper LT, Givertz MM, Alexis JD, Briller J (2017). Myocardial damage detected by late gadolinium enhancement cardiac magnetic resonance is uncommon in Peripartum Cardiomyopathy. J Am Heart Assoc Cardiovasc Cerebrovasc Dis..

[209] Liang Y-D, Xu Y-W, Li W-H, Wan K, Sun J-Y, Lin J-Y (2020). Left ventricular function recovery in peripartum cardiomyopathy: a cardiovascular magnetic resonance stud. J Cardiovasc Magn Reson.

[210] United States Renal Data System. 2020 USRDS Annual data report: epidemiology of kidney disease in the United States. Bethesda, MD: National Institutes of Health, National Institute of Diabetes and Digestive and Kidney Diseases; 2020. https://adr.usrds.org/. Accessed 30 Dec 2020.

[211] Kotecha T, Martinez-Naharro A, Yoowannakul S, Lambe T, Rezk T, Knight DS (2019). Acute changes in cardiac structural and tissue characterisation parameters following haemodialysis measured using cardiovascular magnetic resonance. Sci Rep.

[212] Biersmith MA, Tong MS, Guha A, Simonetti OP, Addison D (2020). Multimodality cardiac imaging in the era of emerging cancer therapies. J Am Heart Assoc Cardiovasc Cerebrovasc Dis..

[213] Lustberg MB, Reinbolt R, Addison D, Ruppert AS, Moore S, Carothers S (2019). Early detection of anthracycline-induced cardiotoxicity in breast cancer survivors with T2 cardiac magnetic resonance. Circ Cardiovasc Imaging.

[214] Martin-Garcia A, Diaz-Pelaez E, Lopez-Corral L, Sanchez-Pablo C, Macias de Plasencia G, Galan-Arriola C (2020). T2 mapping identifies early anthracycline-induced cardiotoxicity in elderly patients with cancer. JACC Cardiovasc Imaging.

[215] Haslbauer JD, Lindner S, Valbuena-Lopez S, Zainal H, Zhou H, D’Angelo T (2019). CMR imaging biosignature of cardiac involvement due to cancer-related treatment by T1 and T2 mapping. Int J Cardiol.

[216] Thavendiranathan P, Amir E, Bedard P, Crean A, Paul N, Nguyen ET (2014). Regional myocardial edema detected by T2 mapping is a feature of cardiotoxicity in breast cancer patients receiving sequential therapy with anthracyclines and trastuzumab. J Cardiovasc Magn Reson.

[217] Ganatra S, Carver JR, Hayek SS, Ky B, Leja MJ, Lenihan DJ (2019). Chimeric antigen receptor T-cell therapy for cancer and heart. J Am Coll Cardiol.

[218] Escudier M, Cautela J, Malissen N, Ancedy Y, Orabona M, Pinto J (2017). Clinical features, management, and outcomes of immune checkpoint inhibitor-related cardiotoxicity. Circulation.

[219] Rao S, Tseng SY, Pednekar A, Siddiqui S, Kocaoglu M, Fares M (2022). Myocardial parametric mapping by cardiac magnetic resonance imaging in pediatric cardiology and congenital heart disease. Circ Cardiovasc Imaging.

[220] Cornicelli MD, Rigsby CK, Rychlik K, Pahl E, Robinson JD (2019). Diagnostic performance of cardiovascular magnetic resonance native T1 and T2 mapping in pediatric patients with acute myocarditis. J Cardiovasc Magn Reson.

[221] Barczuk-Falęcka M, Małek ŁA, Werys K, Roik D, Adamus K, Brzewski M (2020). Normal values of native T1 and T2 relaxation times on 3T cardiac MR in a healthy pediatric population aged 9–18 years. J Magn Reson Imaging JMRI.

[222] Alsaied T, Tseng SY, Siddiqui S, Patel P, Khoury PR, Crotty EJ (2021). Pediatric myocardial T1 and T2 value associations with age and heart rate at 1.5 T. Pediatr Cardiol.

[223] Isaak A, Bischoff LM, Faron A, Endler C, Mesropyan N, Sprinkart AM (2021). Multiparametric cardiac magnetic resonance imaging in pediatric and adolescent patients with acute myocarditis. Pediatr Radiol.

[224] Wang H, Zhao B, Yang H, Qian T, Han B, Jia H (2020). Identifying myocardial injuries in “normal-appearing” myocardium in pediatric patients with clinically suspected myocarditis using mapping techniques. PeerJ.

[225] Sethi N, Doshi A, Doshi T, Cross R, Cronin I, Amin E (2020). Quantitative cardiac magnetic resonance T2 imaging offers ability to non-invasively predict acute allograft rejection in children. Cardiol Young.

[226] Husain N, Watanabe K, Berhane H, Gupta A, Markl M, Rigsby CK (2021). Multi-parametric cardiovascular magnetic resonance with regadenoson stress perfusion is safe following pediatric heart transplantation and identifies history of rejection and cardiac allograft vasculopathy. J Cardiovasc Magn Reson.

